# Supplementary material for: RNA-triggered Cas12a3 cleaves tRNA tails to execute bacterial immunity
Source: Nature. 2026 Jan 7;649(8099):1312–21. doi: 10.1038/s41586-025-09852-9 (PMC12851939; doi:10.1038/s41586-025-09852-9)
Supplement: Supplementary file 1 — This file contains Supplementary Figs. 1–18 and their associated legends. [file 41586_2025_9852_MOESM1_ESM.pdf]

nature portfolio

https://doi.org/10.1038/s41586-025-09852-9

Supplementary information

RNA-triggered Cas12a3 cleaves tRNA tails to execute bacterial immunity

In the format provided by the authors and unedited

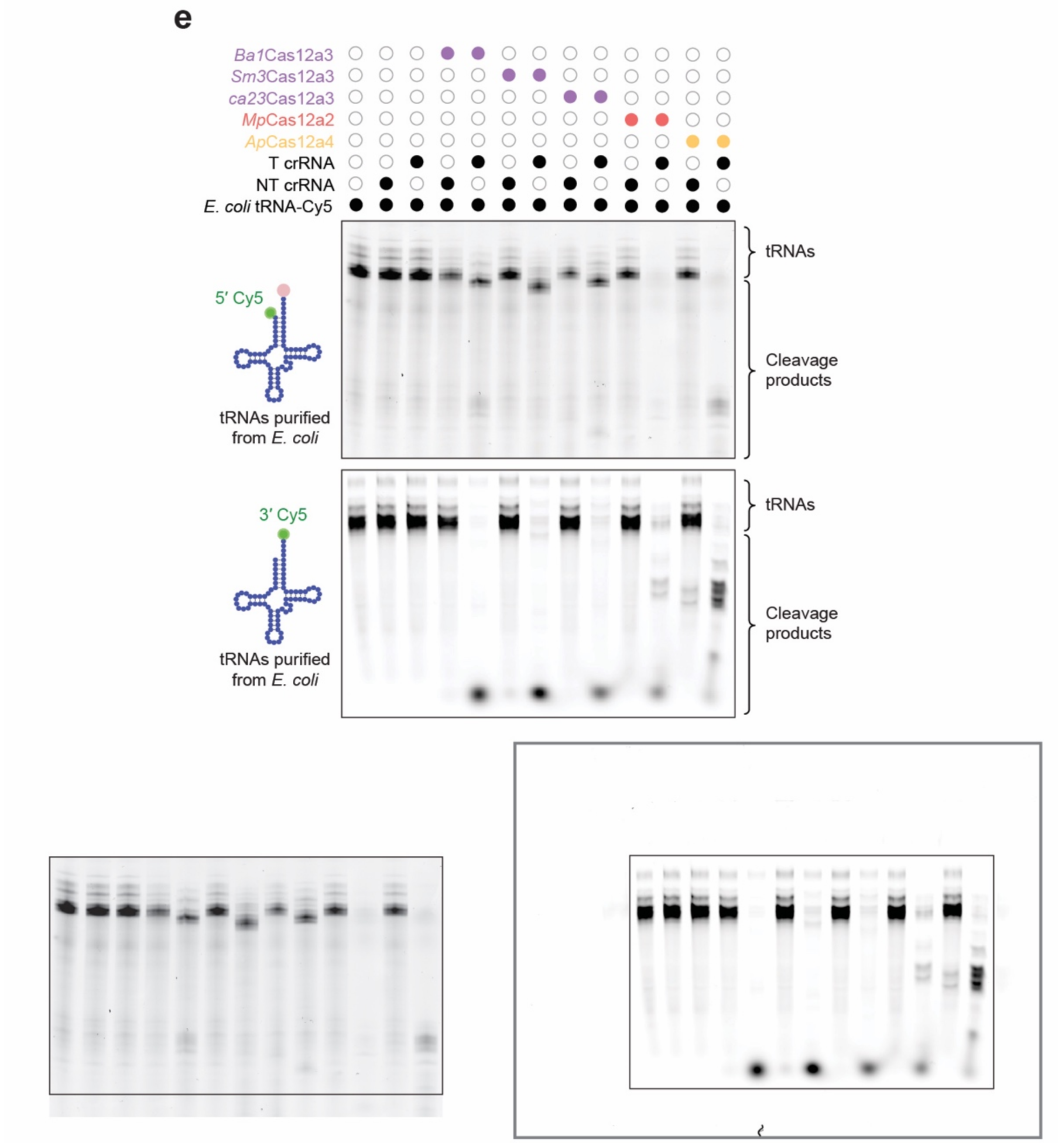

Supplementary Fig. 1. Original gel images. Original gels shown for Figure 2e.

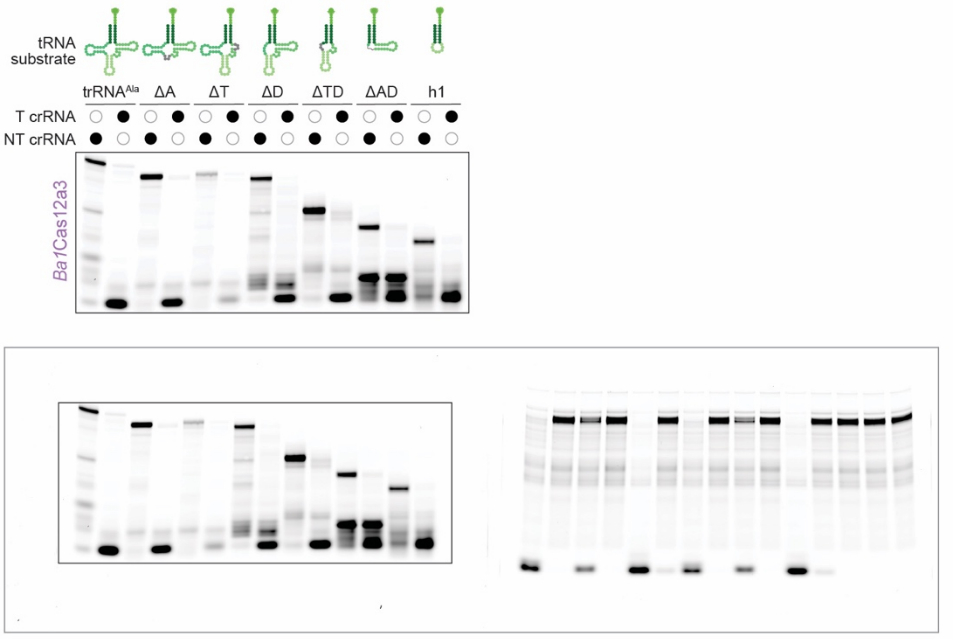

Supplementary Fig. 1. Original gel images cont'd. Original gel shown for Figure 3e.

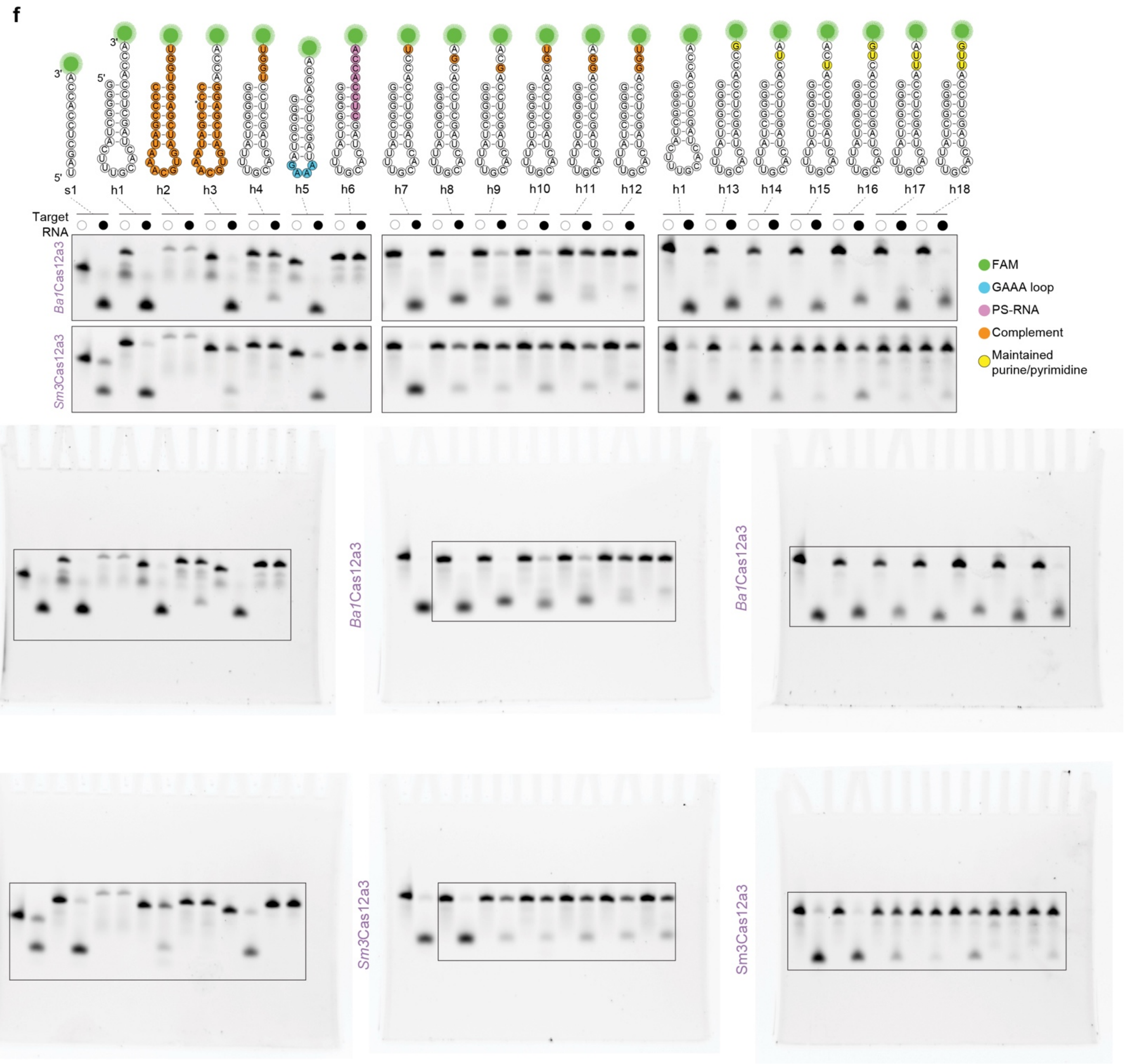

Supplementary Fig. 1. Original gel images cont'd. Original gels shown for Figure 3f.

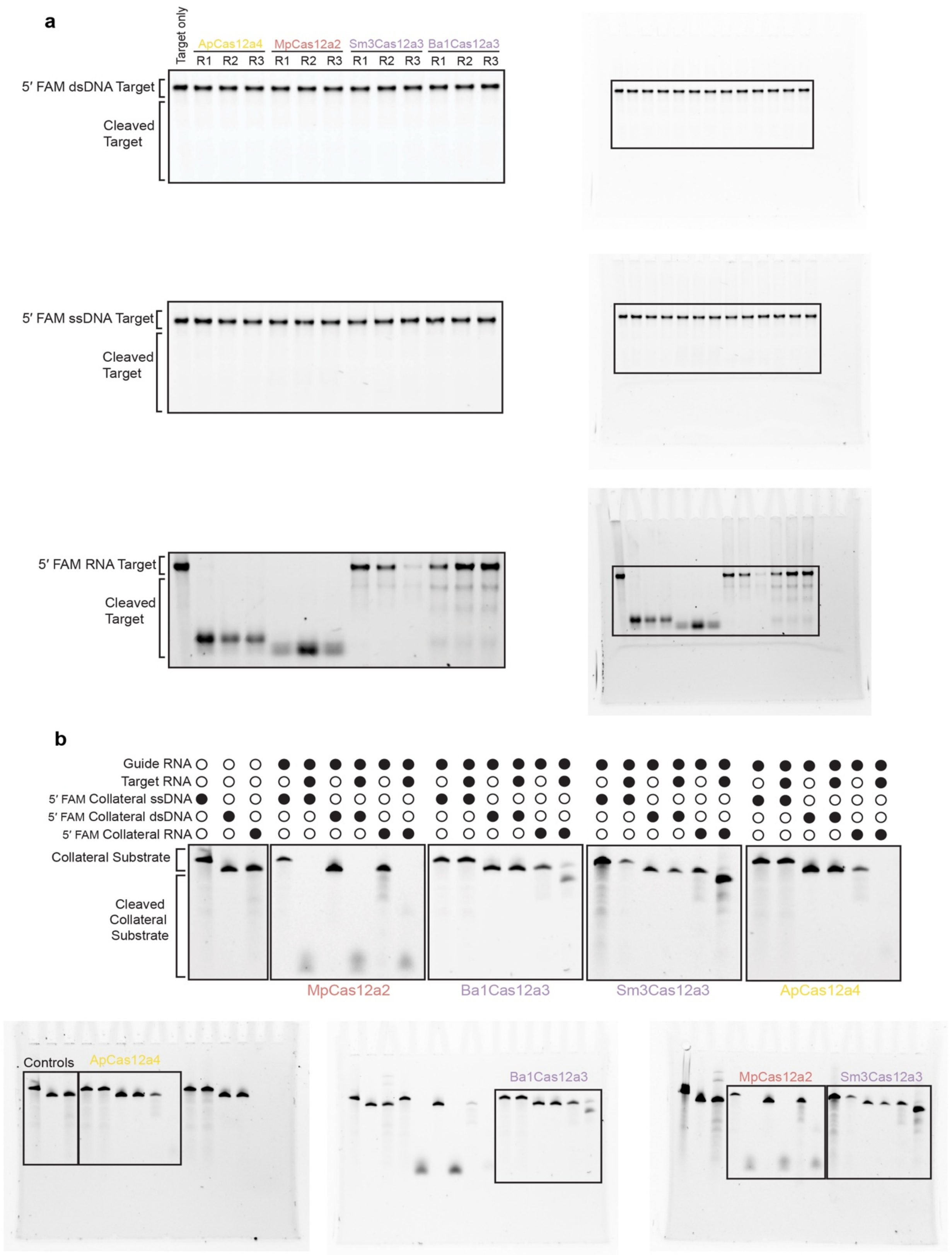

Supplementary Fig. 1. Original gel images cont'd. Original gels shown for Extended Data Figure 2a,b.

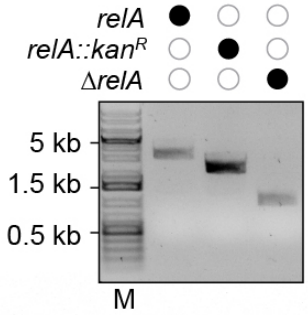

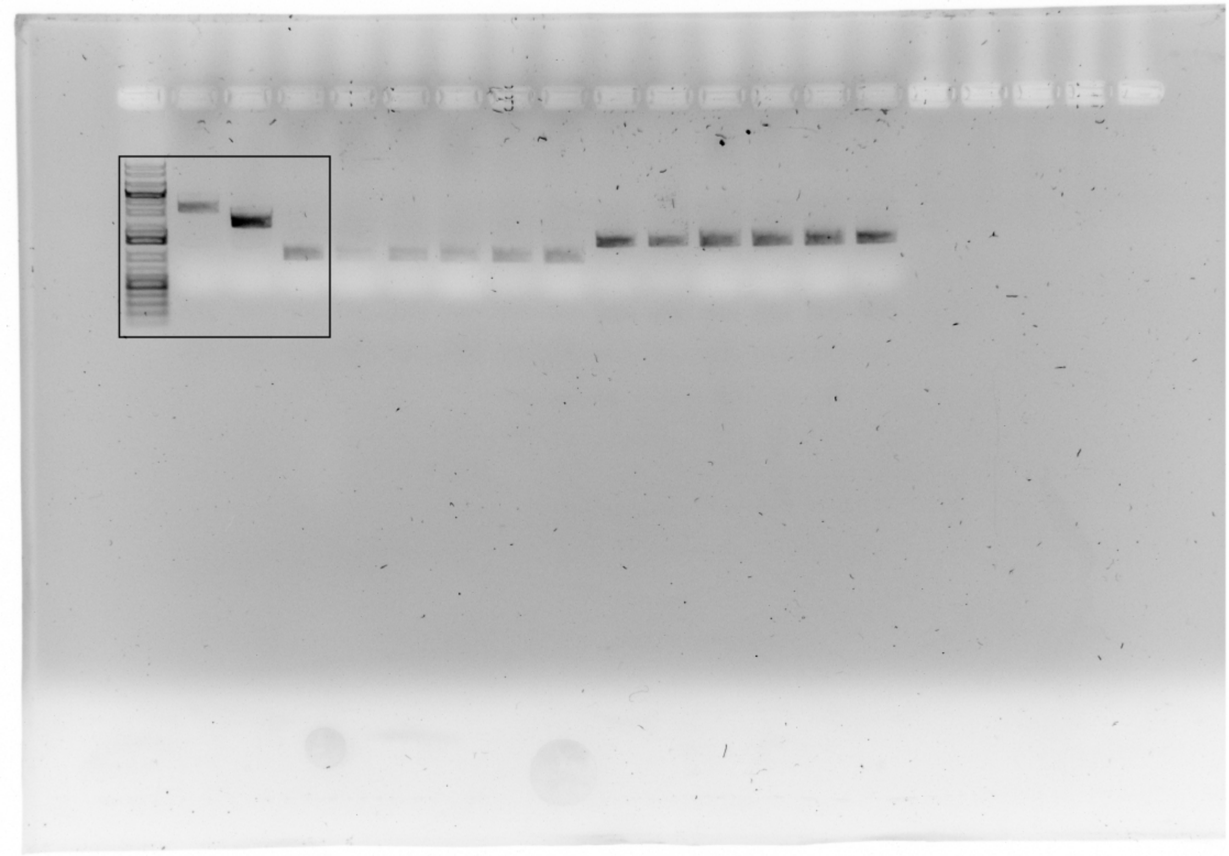

Supplementary Fig. 1. Original gel images cont'd. Original gels shown for Extended Data Figure 4a.

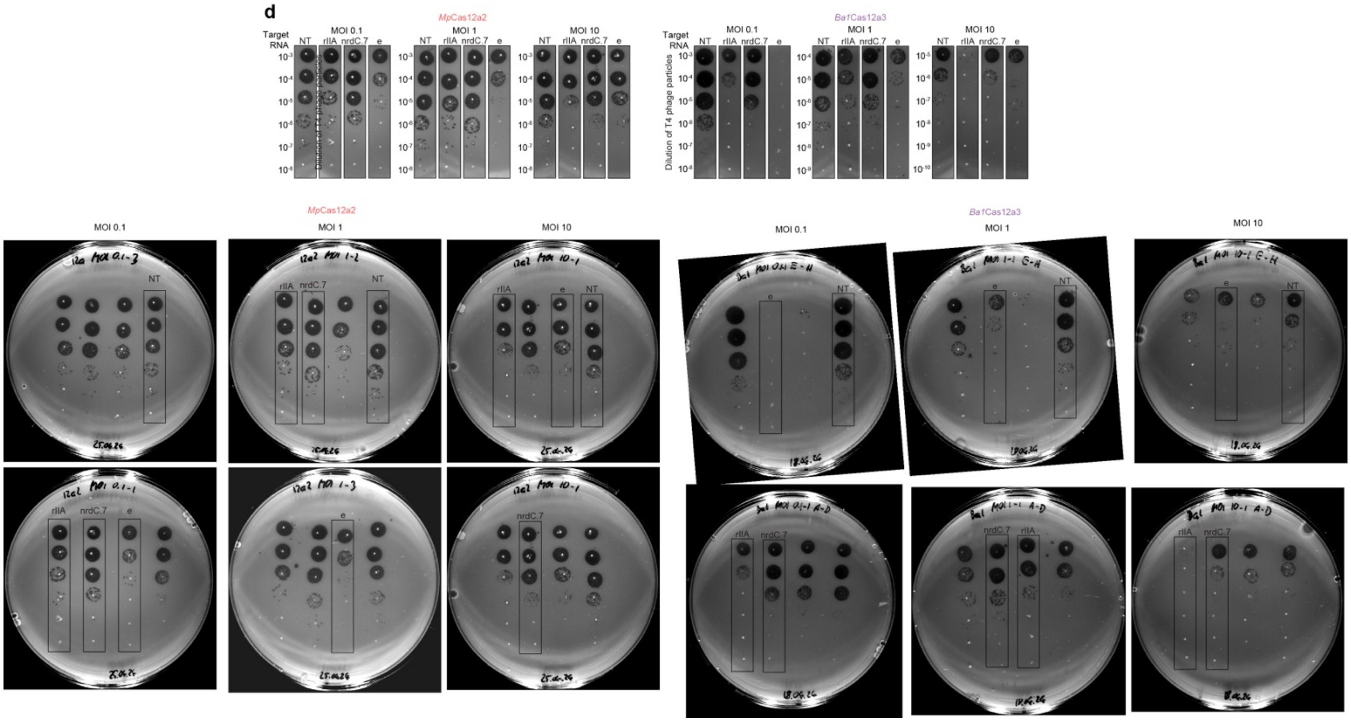

Supplementary Fig. 1. Original gel images cont'd. Original gels shown for Supplementary Figure 2d.

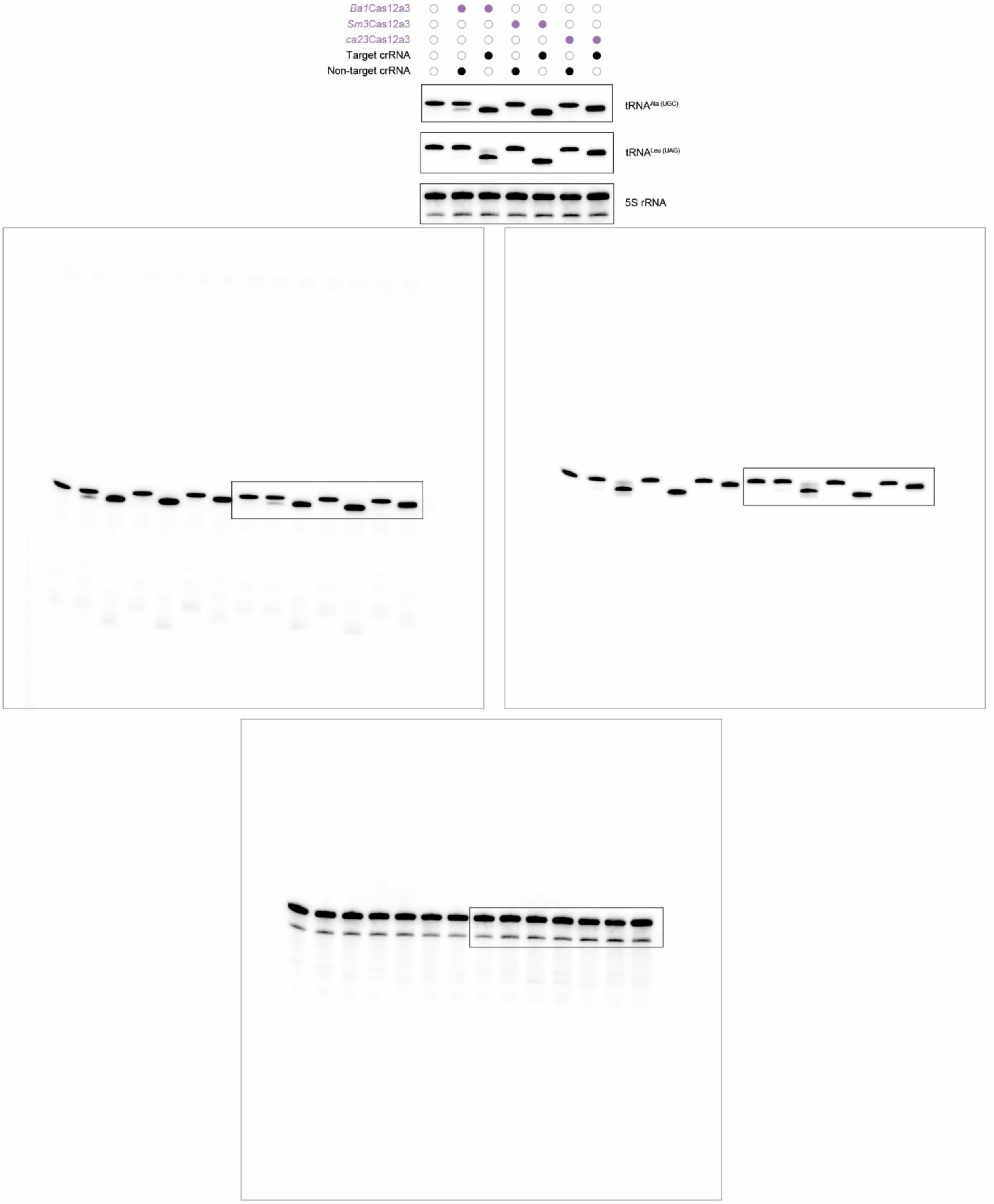

Supplementary Fig. 1. Original gel images cont'd. Original gels shown for Supplementary Figure 4b.

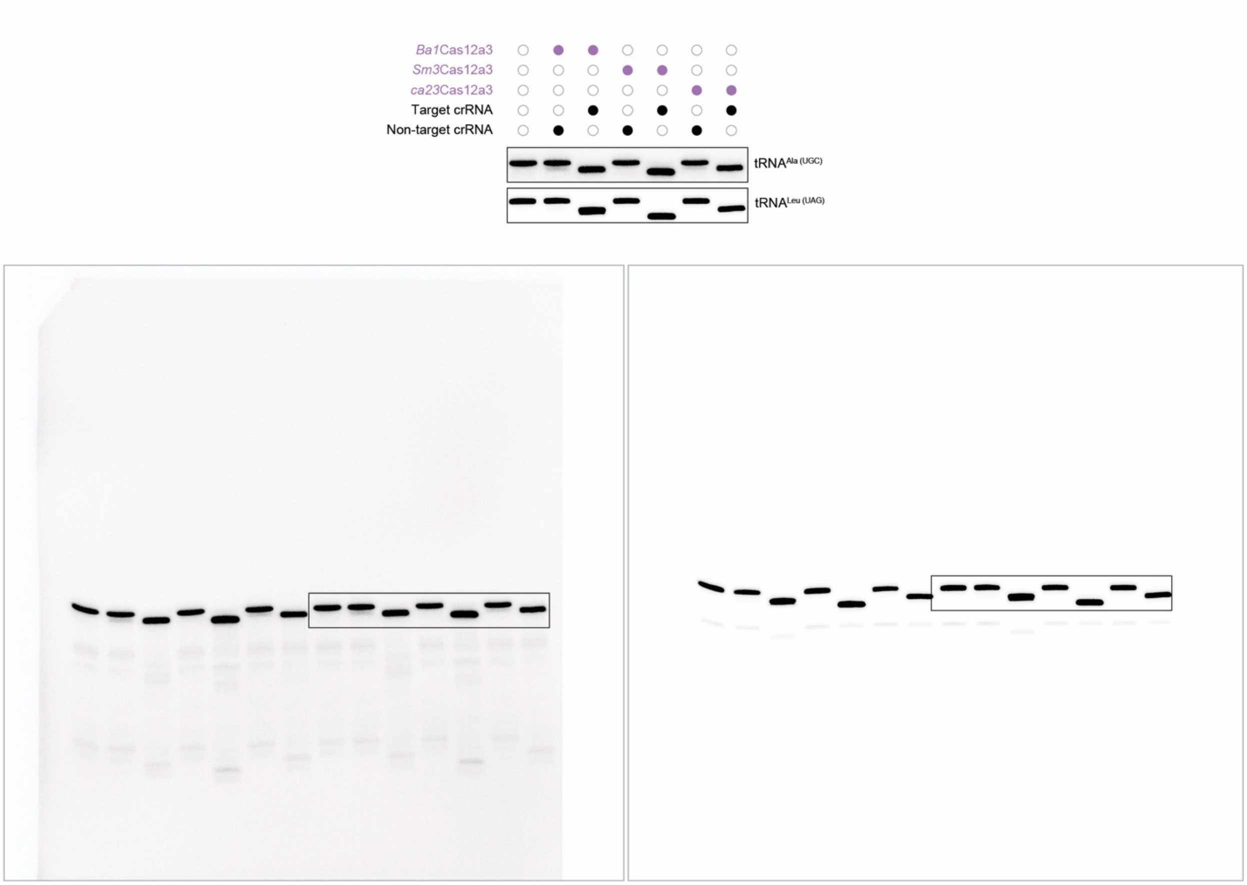

Supplementary Fig. 1. Original gel images cont'd. Original gels shown for Supplementary Figure 6.

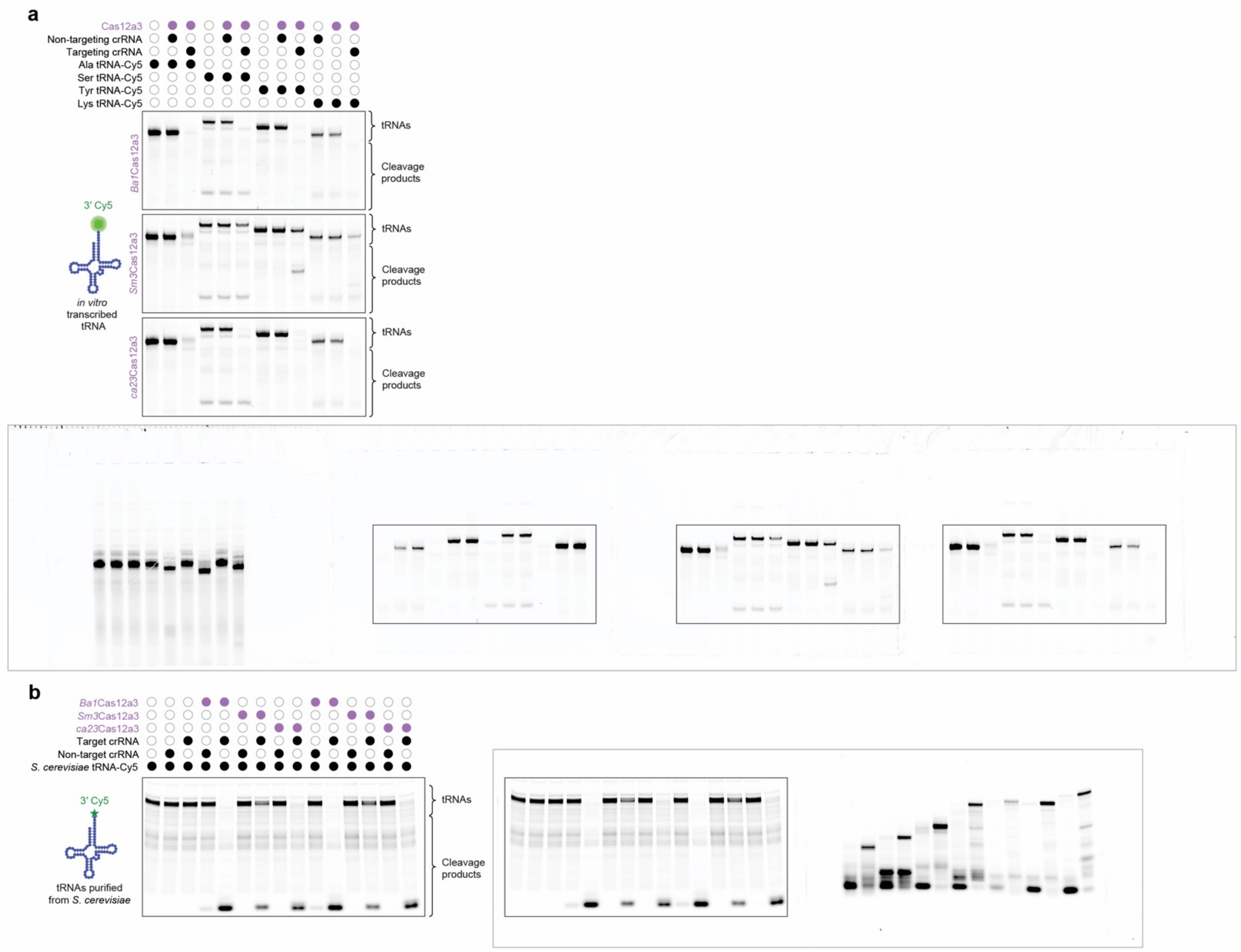

Supplementary Fig. 1. Original gel images cont'd. Original gels shown for Supplementary Figure 7a,b.

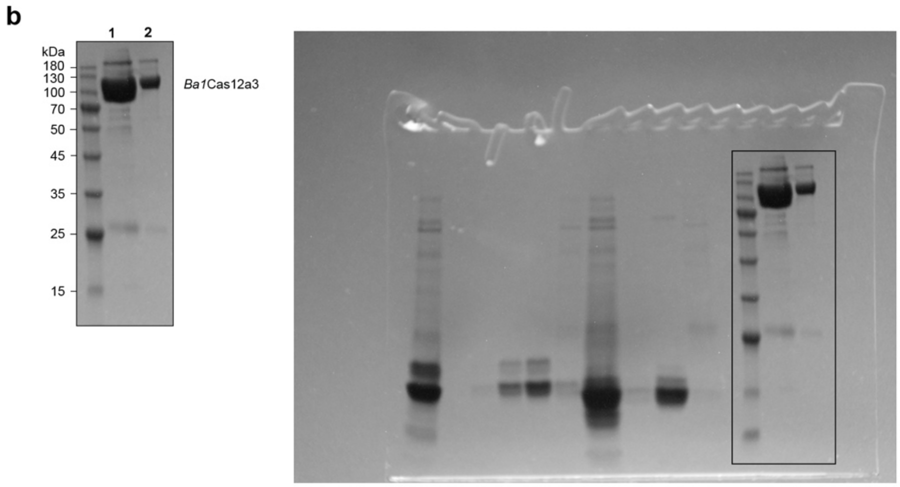

Supplementary Fig. 1. Original gel images cont'd. Original gels shown for Supplementary Figure 8b.

b

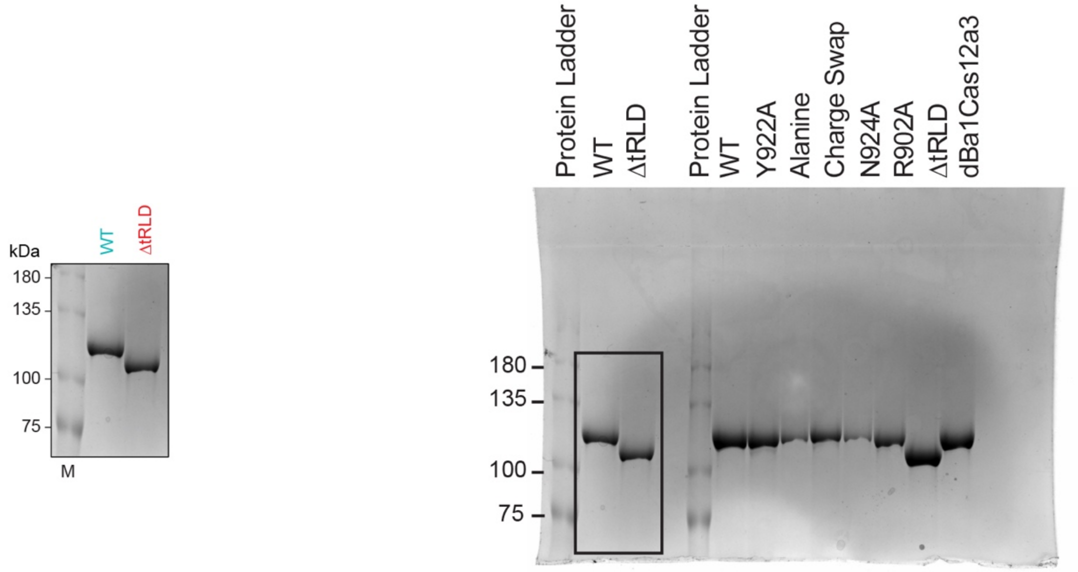

d

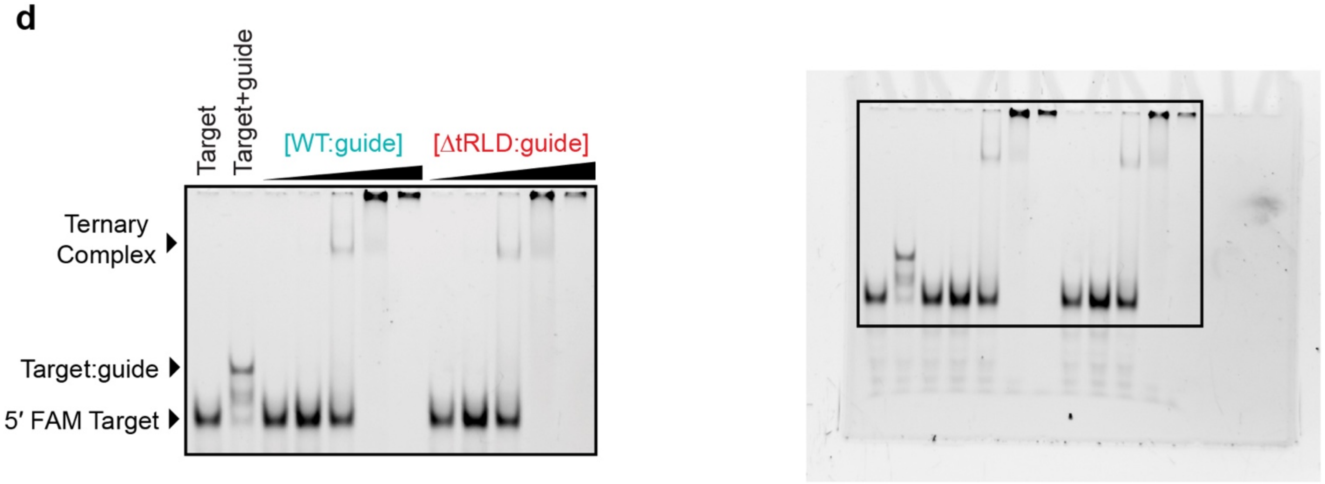

Supplementary Fig. 1. Original gel images cont'd. Original gels shown for Supplementary Figure 12b,d.

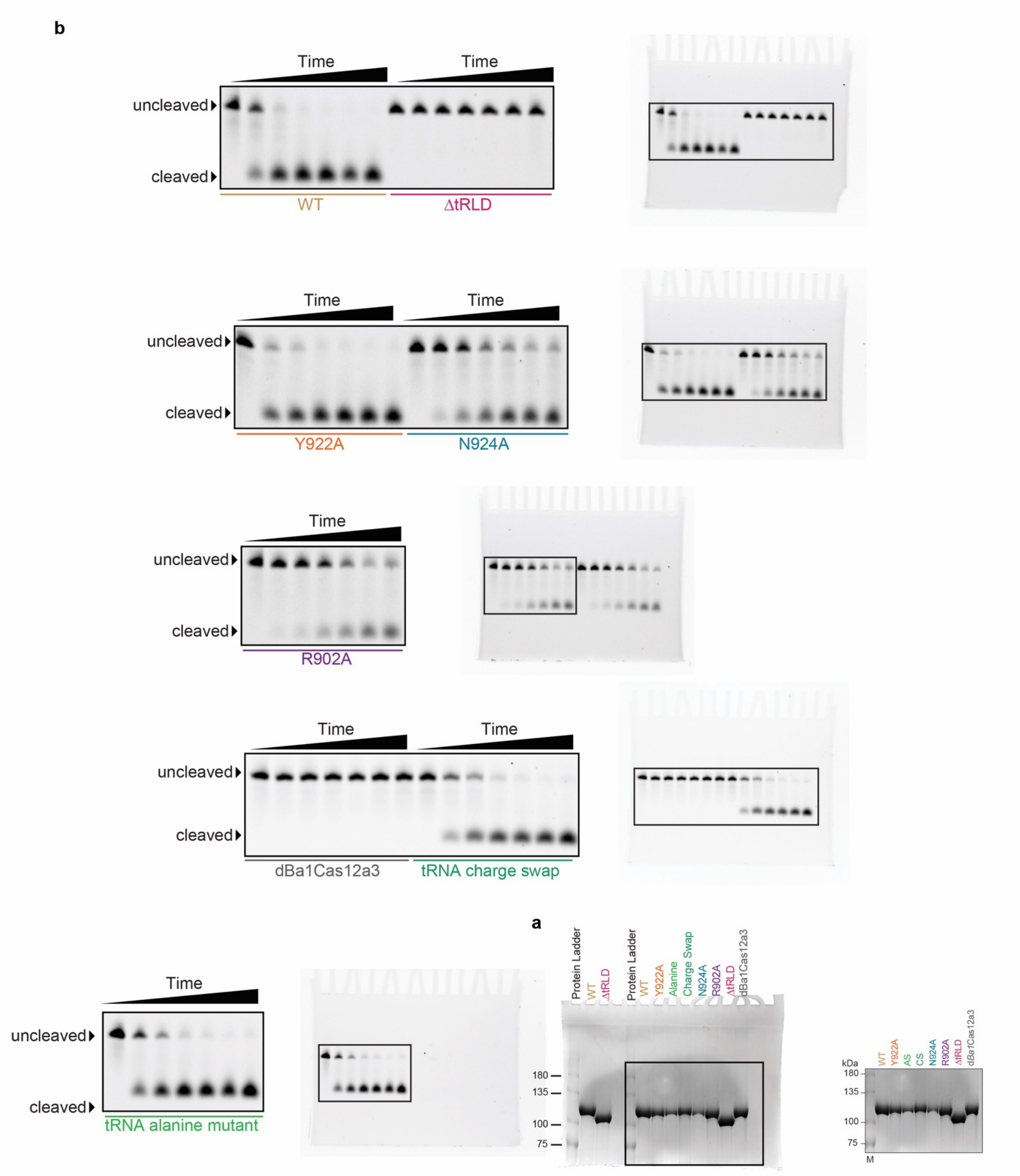

Supplementary Fig. 1. Original gel images cont'd. Original gels shown for Supplementary Figure 14a,b.

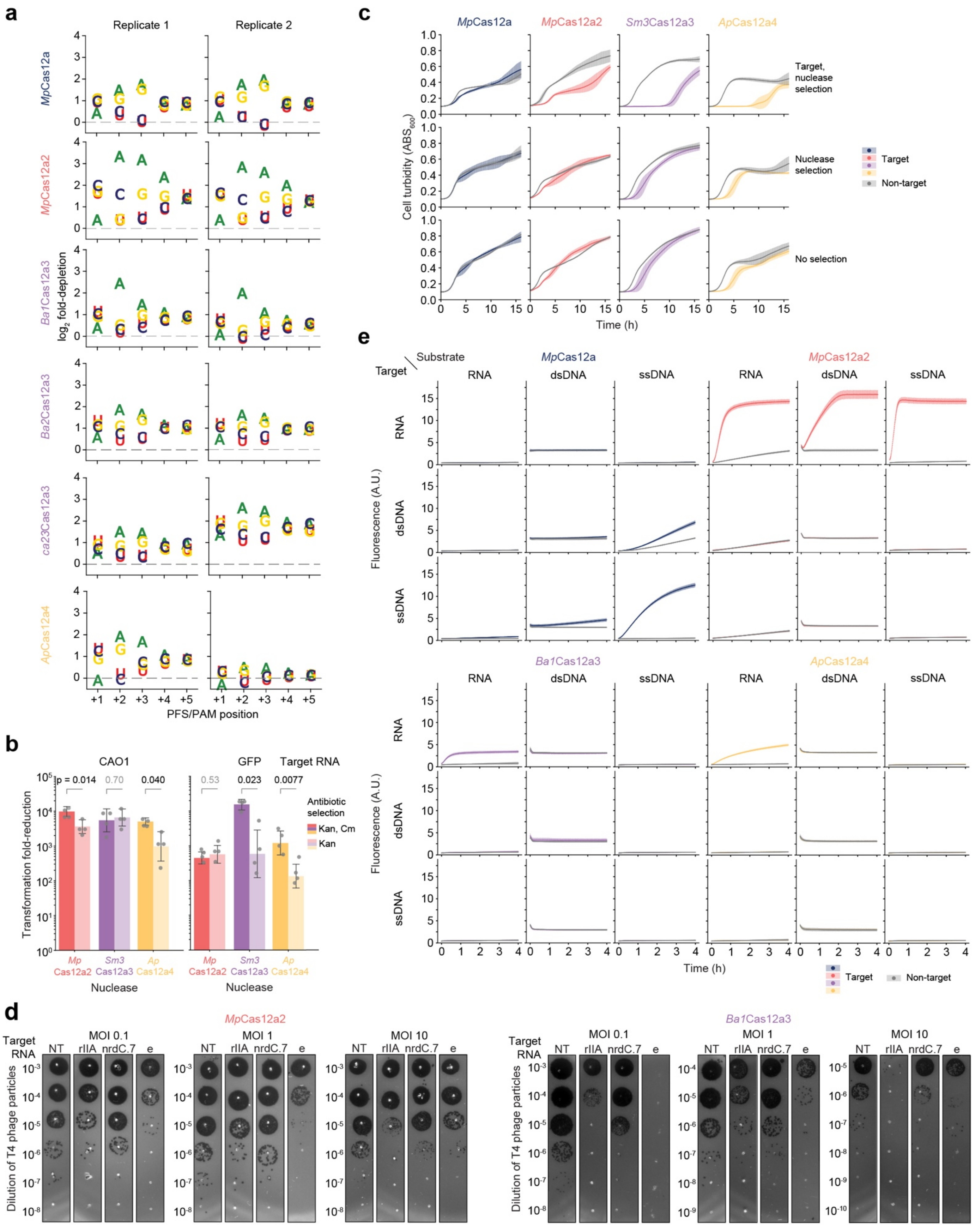
a
Replicate 1
Replicate 2
MpCas12a
MpCas12a2
Ba1Cas12a3
Ba2Cas12a3
ca23Cas12a3
ApCas12a4
log2 fold-depletion
PFS/PAM position
b
CAO1
GFP
Target RNA
p = 0.014
0.70
0.040
0.53
0.023
0.0077
Transformation fold-reduction
Antibiotic selection
Kan, Cm
Kan
Mp Cas12a2
Sm3 Cas12a3
Ap Cas12a4
Nuclease
c
MpCas12a
MpCas12a2
Sm3Cas12a3
ApCas12a4
Cell turbidity (ABS600)
Target, nuclease selection
Nuclease selection
No selection
Target
Non-target
Time (h)
d
MpCas12a2
Ba1Cas12a3
Target RNA
MOI 0.1
MOI 1
MOI 10
NT
rIIA
nrdC.7
e
Dilution of T4 phage particles
e
Substrate
Target
RNA
dsDNA
ssDNA
Fluorescence (A.U.)
Time (h)
Target
Non-target

Supplementary Fig. 2. Extended characterization of Cas12a3 and Cas12a4 nucleases in *E. coli* and *in vitro*. (a) Nucleotide depletion plots following the screen of the 5-nucleotide PFS/PAM library. Note that the PAM for *Mp*Cas12a is the reverse complement of the sequence normally reported for Cas12a nucleases (*i.e.,* YYV). Each plot shows the mean of independent screens in *E. coli* (n = 2). The PFS screen for *Sm3*Cas12a3 is not shown due to insufficient sequence coverage. **(b)** Plasmid interference assay in *E. coli* targeting CAO1 and GFP transcripts. See **Figure 1d** for a diagram of the assay. Plasmid clearance and growth arrest are differentiated based on selection for the target plasmid. Bars and error bars represent the geometric mean ± geometric standard deviation of independent experiments starting from separate colonies, with gray dots representing each measurement (n = 4). **(c)** Growth curves (OD_{600}) of *E. coli* expressing each nuclease (*Mp*Cas12a, *Mp*Cas12a2, *Sm3*Cas12a3, and *Ap*Cas12a4), with either target or non-target crRNAs and target RNA. Expression was induced at t = 0 under the indicated selection conditions. Scatter plots with error bars represent the mean ± standard deviation of independent experiments (n = 4) started from separate colonies. **(d)** Protection conferred by *Mp*Cas12a2 and *Ba1*Cas12a3 against T4 phage in *E. coli*. Bacterial cultures of *E. coli* carrying nuclease expression plasmids, alongside plasmids encoding either a non-target (NT) crRNA or a crRNA targeting T4 transcripts, were infected with T4 at the indicated multiplicities of infection (MOIs) and grown overnight. The following day, the phages in these cultures were quantified with a plaque assay. The plaques illustrate the degree of protection conferred by the nucleases. Images are representative of independent experiments (n = 3) starting from separate colonies. **(e)** *In vitro* collateral substrate cleavage in response to different target types. Scatter plots and error bars show the mean ± standard deviation of independent reactions (n = 3 or 4). The corresponding initial linear rates, normalized per each nuclease, are shown in **Figure 1f**. Statistical analyses were performed using two-tailed Welch’s t-tests. P-values that are not significant ($p \geq 0.05$) are shown in gray.

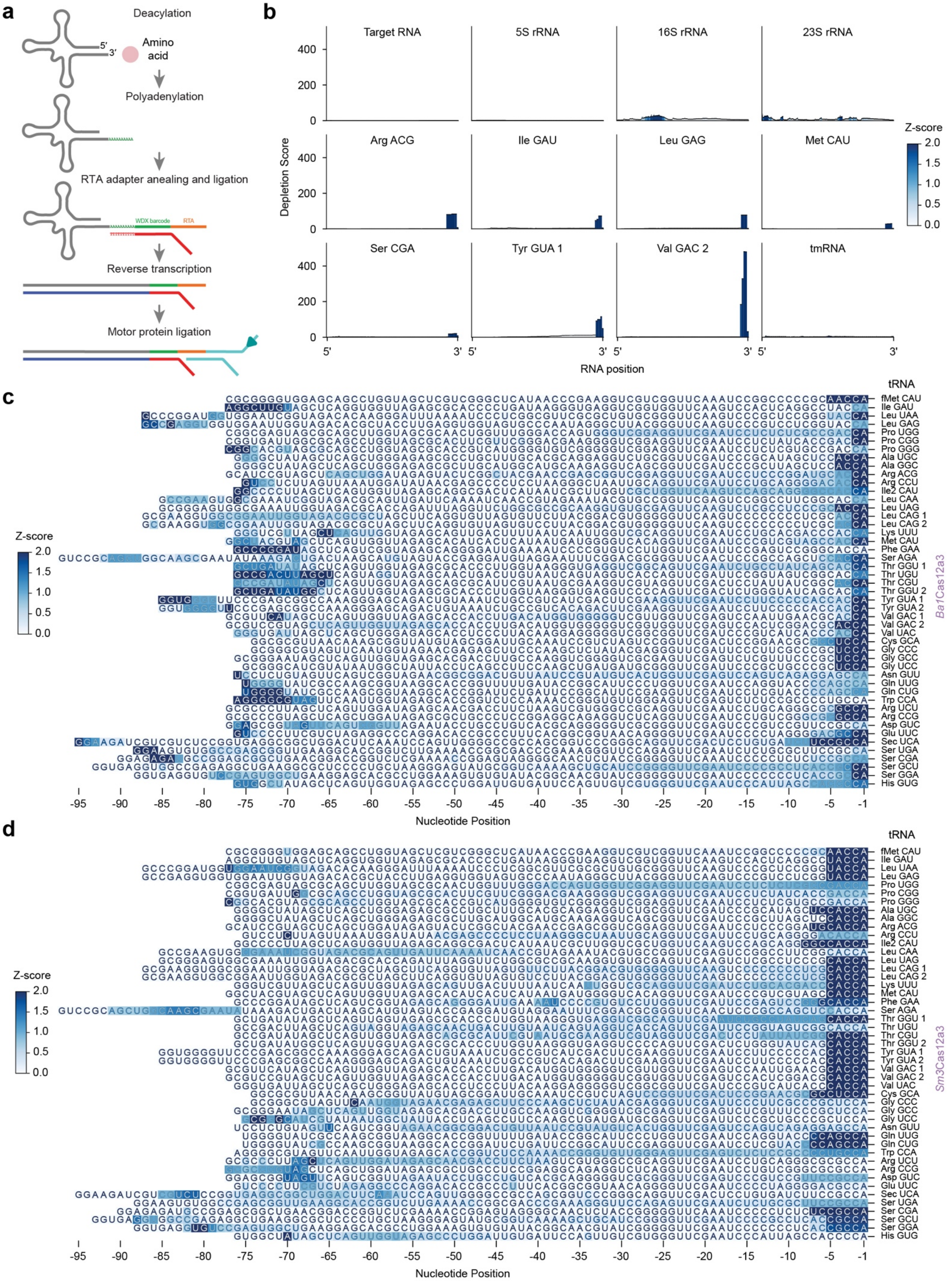
a
Deacylation
5′
3′
Amino acid
Polyadenylation
RTA adapter annealing and ligation
WDX barcode
RTA
Reverse transcription
Motor protein ligation
b
Target RNA
5S rRNA
16S rRNA
23S rRNA
Arg ACG
Ile GAU
Leu GAG
Met CAU
Ser CGA
Tyr GUA 1
Val GAC 2
tmRNA
Depletion Score
RNA position
Z-score
c
tRNA
Ba1Cas12a3
Nucleotide Position
d
tRNA
Sm3Cas12a3
Nucleotide Position

Supplementary Fig. 3. Direct RNA Nanopore sequencing of target RNA, rRNA, tmRNA, and tRNA under RNA-target and non-target conditions with *Ba1*Cas12a3 and *Sm3*Cas12a3 in TXTL. (a) Schematic of the RNA sample preparation protocol for direct RNA sequencing via Nanopore, using tRNA as an example. **(b)** Plots showing sequencing coverage depletion scores of selected rRNA, tRNA, and tmRNA at each nucleotide position under non-target (NT) versus target (T) conditions with *Sm3*Cas12a3. Data represent averages from independent TXTL experiments (n = 3), in which RNAs were barcoded with WDX adapters for sequencing and demultiplexed following high-fidelity basecalling. The color map indicates the Z-score (number of standard deviations from the mean) of depletion scores at each position. **(c)** tRNA sequences identified from independent TXTL experiments (n = 3) with *Ba1*Cas12a3 under NT and T conditions, showing NT/T depletion Z-scores for each nucleotide as a color map. **(d)** tRNA sequences identified from independent TXTL experiments (n = 3) with *Sm3*Cas12a3 under NT and T conditions, likewise displayed as NT/T depletion Z-scores at each nucleotide position.

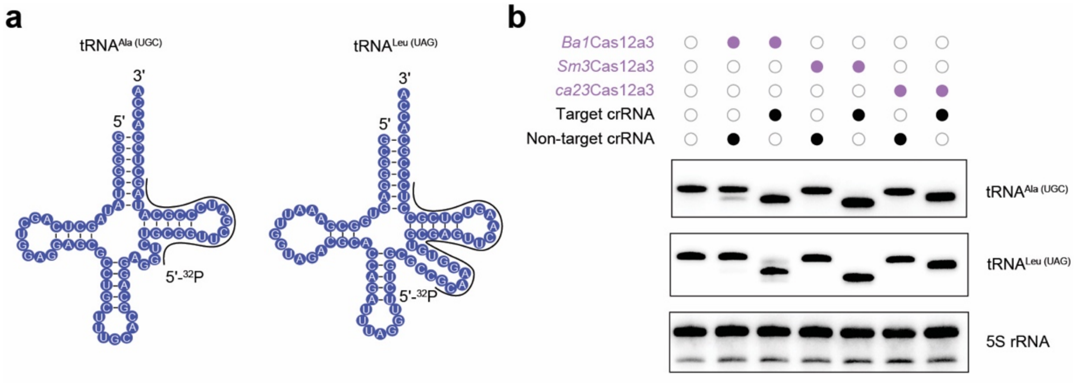

Supplementary Fig. 4. Northern blotting analysis of selected tRNAs cleaved with Cas12a3 in TXTL. (a) Probing of $tRNA^{Ala(UGC)}$ and $tRNA^{Leu(UAG)}$ by northern blotting analysis. The binding location of the oligonucleotide probes are indicated. **(b)** Blots for the two probed tRNAs as well as 5S rRNA. The indicated Cas12a3 RNP was incubated in TXTL, and the extracted total RNA was subjected to northern blotting analysis. Gel images are representative of independent biological replicates (n = 3). For gel source data, see Supplementary Figure 1.

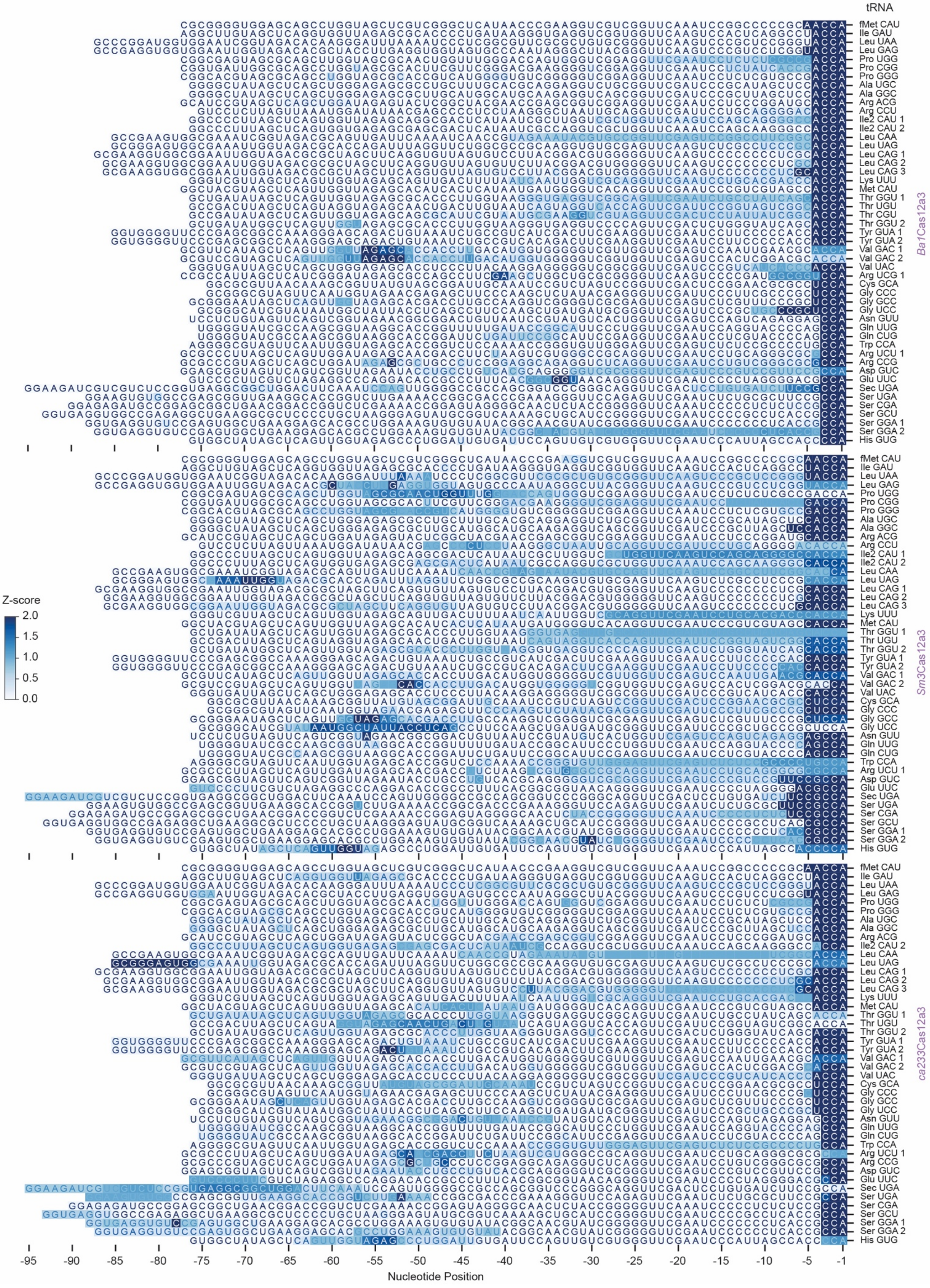
tRNA
Z-score
2.0
1.5
1.0
0.5
0.0
Ba1Cas12a3
Sm3Cas12a3
ca233Cas12a3
-95 -90 -85 -80 -75 -70 -65 -60 -55 -50 -45 -40 -35 -30 -25 -20 -15 -10 -5 -1
Nucleotide Position

Supplementary Fig. 5. Direct RNA Nanopore sequencing of purified tRNA from *E. coli* MRE600 under RNA-target and non-target conditions with *Ba1*Cas12a3 and *Sm3*Cas12a3 *in vitro.* Only the tRNA sequences (labeled with the corresponding anticodons) identified in independent *in vitro* experiments are shown (n = 3). The color map shows NT/T depletion Z-scores for each nucleotide position.

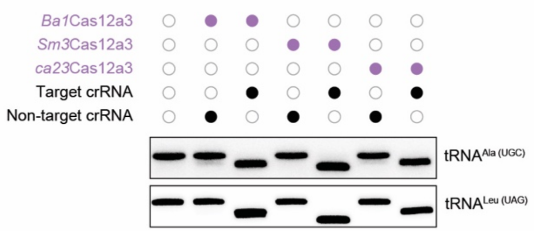

Supplementary Fig. 6. Northern blotting analysis of selected tRNAs cleaved with Cas12a3 *in vitro.* Blots for the two probed tRNAs as well as 5S rRNA. The indicated Cas12a3 RNP was incubated with purified *E. coli* tRNAs *in vitro*, and the extracted total RNA was subjected to northern blotting analysis. Gel images are representative of independent biological replicates (n = 3). See Supplementary Fig. 4a for the probe locations. For gel source data, see Supplementary Figure 1.

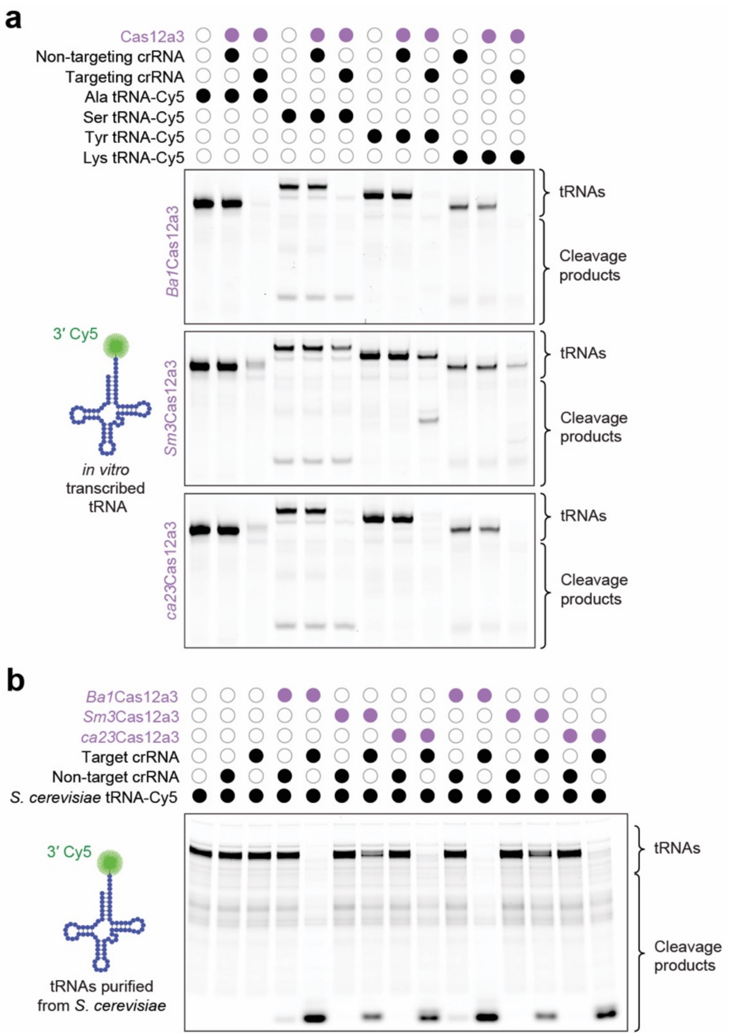

Supplementary Fig. 7. *In vitro* cleavage of *in vitro*-transcribed tRNAs and yeast tRNAs by different Cas12a3 orthologs. (a) Cleavage of *in vitro*-transcribed tRNAs. **(b)** Cleavage of yeast tRNAs. The tRNAs were 3′ labeled with cyanine 5 (Cy5). Gel images are representative of independent cleavage reactions (n = 2 for **a**, n = 3 for **b**). For gel source data, see Supplementary Figure 1.

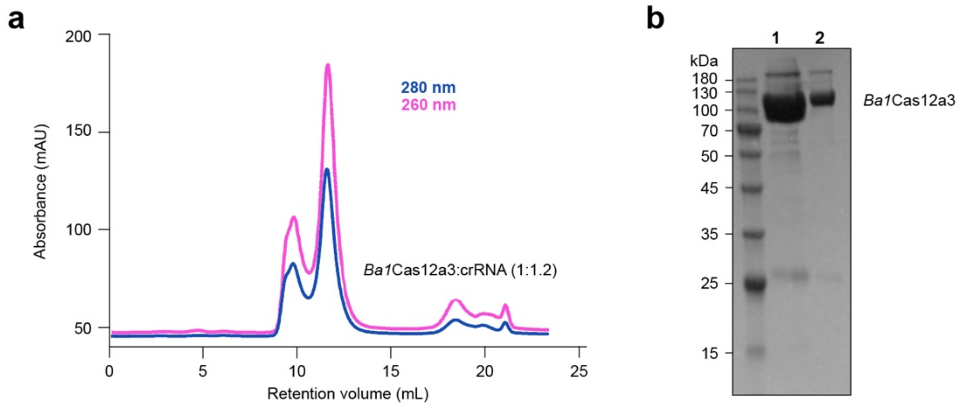

Supplementary Fig. 8. Sample preparation for *Ba1*Cas12a3 binary complex. (a) Size-exclusion chromatography (SEC) profile of *Ba1*Cas12a3 in the presence of crRNA at a molar ratio of 1:1.2. **(b)** SDS-PAGE of purified *Ba1*Cas12a3 binary complex. Lane 1: Sample before SEC; lane 2: Peak fraction from SEC. For gel source data, see Supplementary Figure 1.

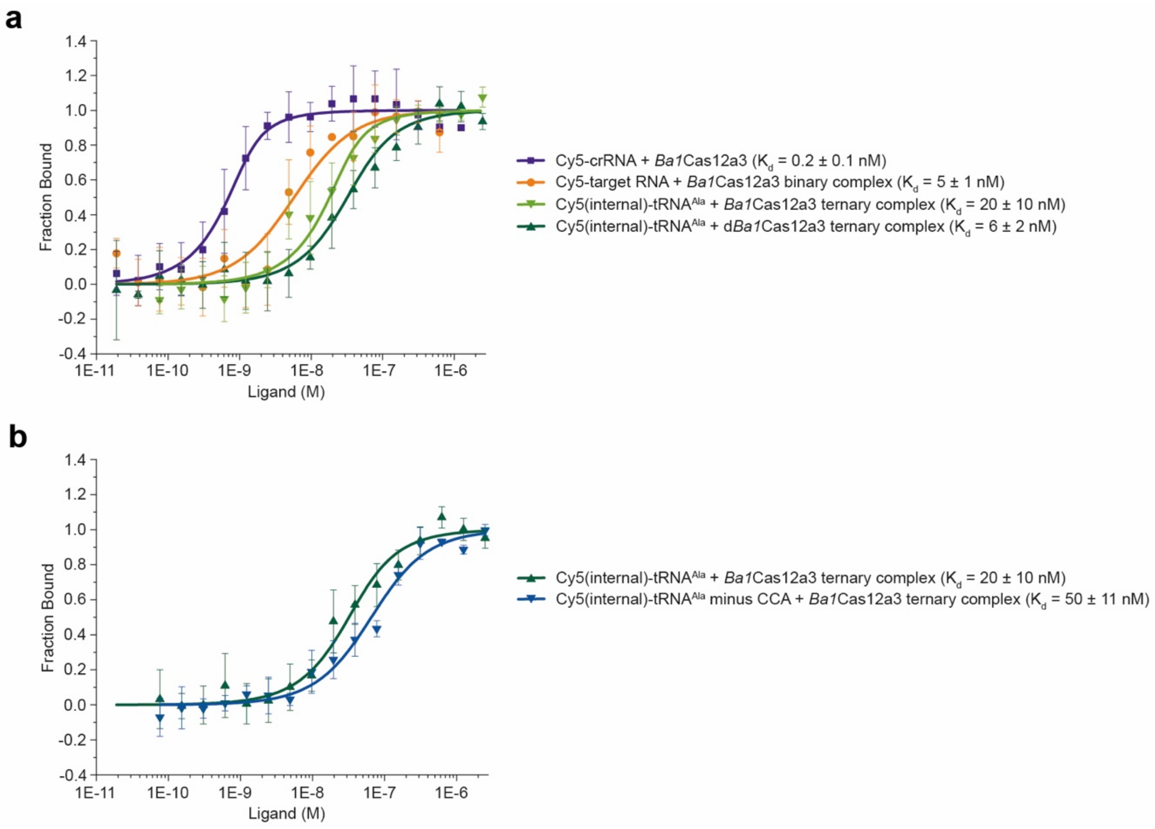

Supplementary Fig. 9. Microscale thermophoresis (MST) measurements of *Ba1*Cas12a3 binding to crRNA, target RNA, and $\mathbf{tRNA^{Ala\ (UGC)}}$. (a) MST-based quantification of *Ba1*Cas12a3 binding to a targeting crRNA, target RNA, and $tRNA^{Ala(UGC)}$. d*Ba1*Cas12a3 contains the E1065A mutation in the RuvC endonuclease domain to render it catalytically inactive. **(b)** *Ba1*Cas12a3 binding of $tRNA^{Ala(UGC)}$ with and without the 3′ CCA tail. Symbols and error bars represent the mean ± standard deviation of independent experiments (n = 3).

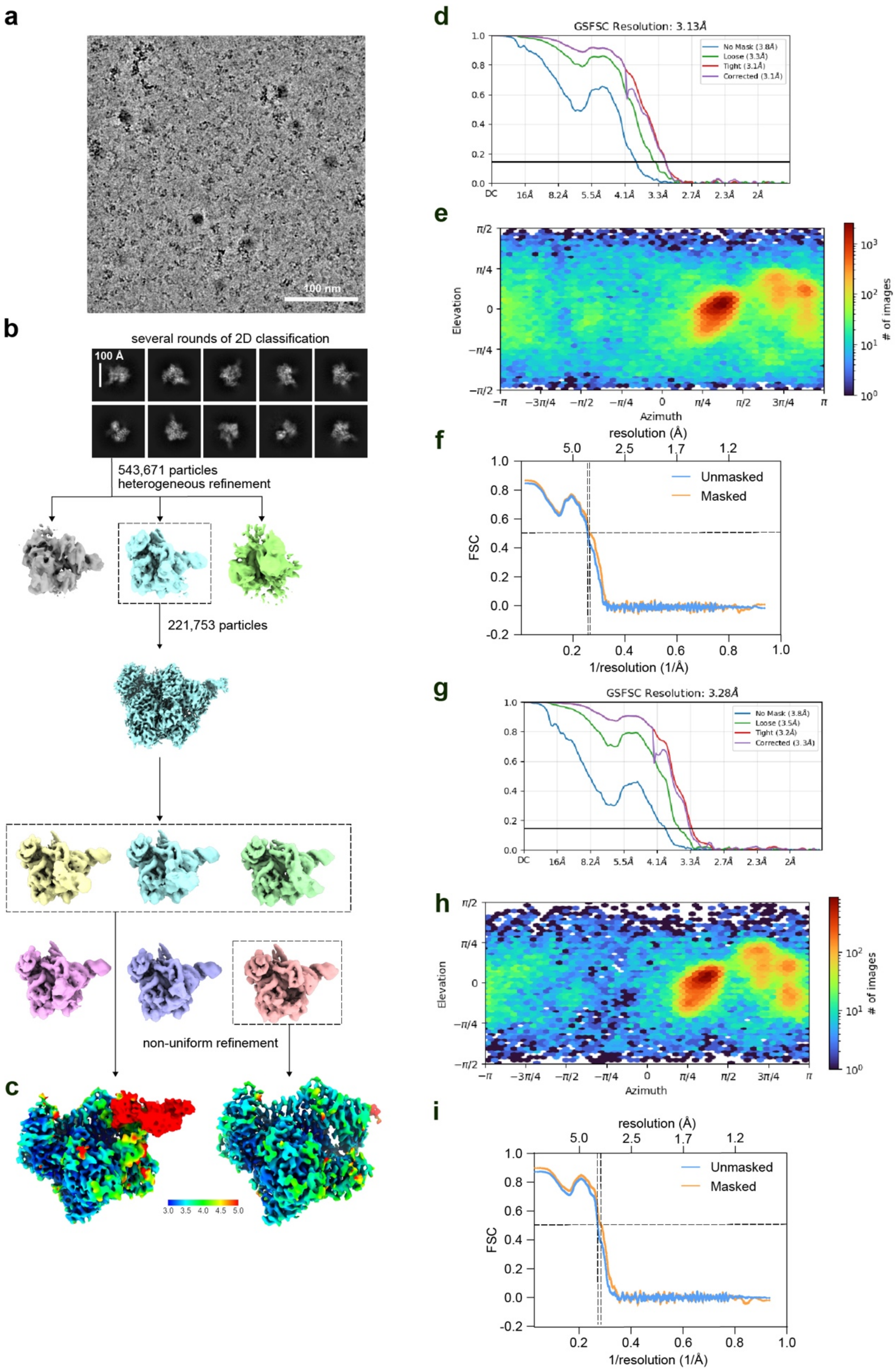
a
100 nm
b
several rounds of 2D classification
100 Å
543,671 particles
heterogeneous refinement
221,753 particles
non-uniform refinement
c
3.0 3.5 4.0 4.5 5.0
d
GSFSC Resolution: 3.13Å
No Mask (3.8Å)
Loose (3.3Å)
Tight (3.1Å)
Corrected (3.1Å)
e
Elevation
Azimuth
# of images
f
resolution (Å)
Unmasked
Masked
FSC
1/resolution (1/Å)
g
GSFSC Resolution: 3.28Å
No Mask (3.8Å)
Loose (3.5Å)
Tight (3.2Å)
Corrected (3.3Å)
h
Elevation
Azimuth
# of images
i
resolution (Å)
Unmasked
Masked
FSC
1/resolution (1/Å)

Supplementary Fig. 10. Data processing scheme of *Ba1*Cas12a3 quaternary complexes. (a) Representative cryo-EM micrograph of *Ba1*Cas12a3 quaternary complexes. **(b)** Data processing scheme for *Ba1*Cas12a3 quaternary complexes at pre-cleavage and post-cleavage stages. **(c)** Cryo-EM structure of *Ba1*Cas12a3 quaternary complexes colored by local spatial resolution in Å (left panel: pre-cleavage stage; right panel: post-cleavage stage). **(d)** Gold standard Fourier Shell Correlation (FSC) curves for *Ba1*Cas12a2 quaternary complex at pre-cleavage stage. **(e)** Euler plot for *Ba1*Cas12a3 quaternary complex at pre-cleavage stage. **(f)** Map-to-model FSC curves for *Ba1*Cas12a3 quaternary complex at pre-cleavage stage. **(g)** FSC curves for *Ba1*Cas12a2 quaternary complex at post-cleavage stage. **(h)** Euler plot for *Ba1*Cas12a3 quaternary complex at the post-cleavage stage. **(i)** Map-to-model FSC curves for *Ba1*Cas12a3 quaternary complex at post-cleavage stage.

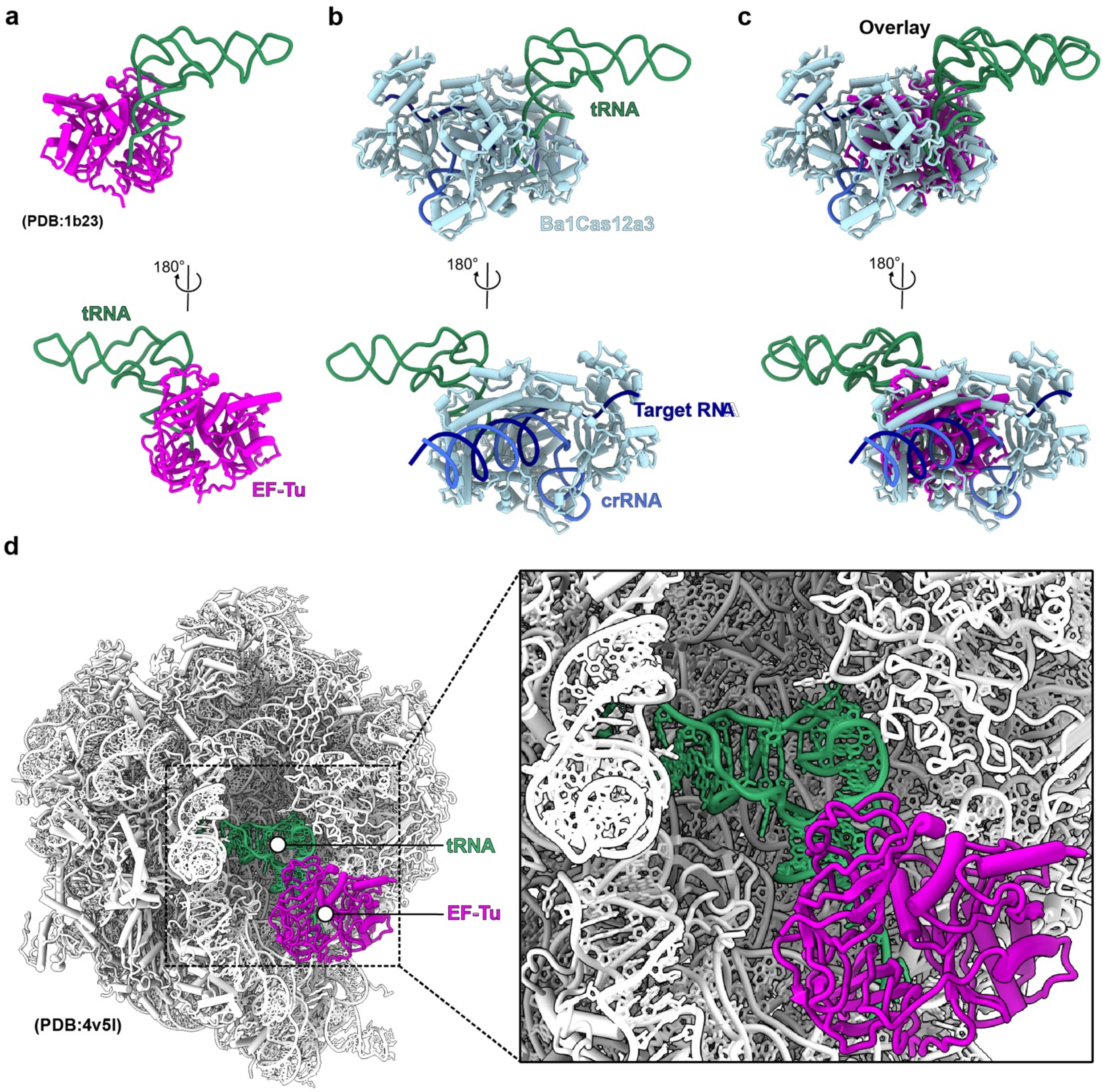

Supplementary Fig. 11. Structural comparison between the Ba1Cas12a3 quaternary complex and the EF-Tu-tRNA complex. (a) Structure of the EF-Tu-tRNA complex (PDB ID: 1b23). EF-Tu is shown in magenta and tRNA in green. **(b)** Structure of the *Ba1*Cas12a3 quaternary complex. *Ba1*Cas12a3 is shown in light blue, tRNA in green, crRNA in royal blue and the target RNA in dark blue. **(c)** Superimposition of the *Ba1*Cas12a3 quaternary complex and the EF-Tu-tRNA complex, showing massive steric clashes between both proteins. The structures

were aligned on tRNA. The structures were colored as in panels a and b. **(d)** Structure of EF-Tu-tRNA bound to the ribosome (PDB ID: 4v5l), showing the tRNA being deeply buried inside the ribosome.

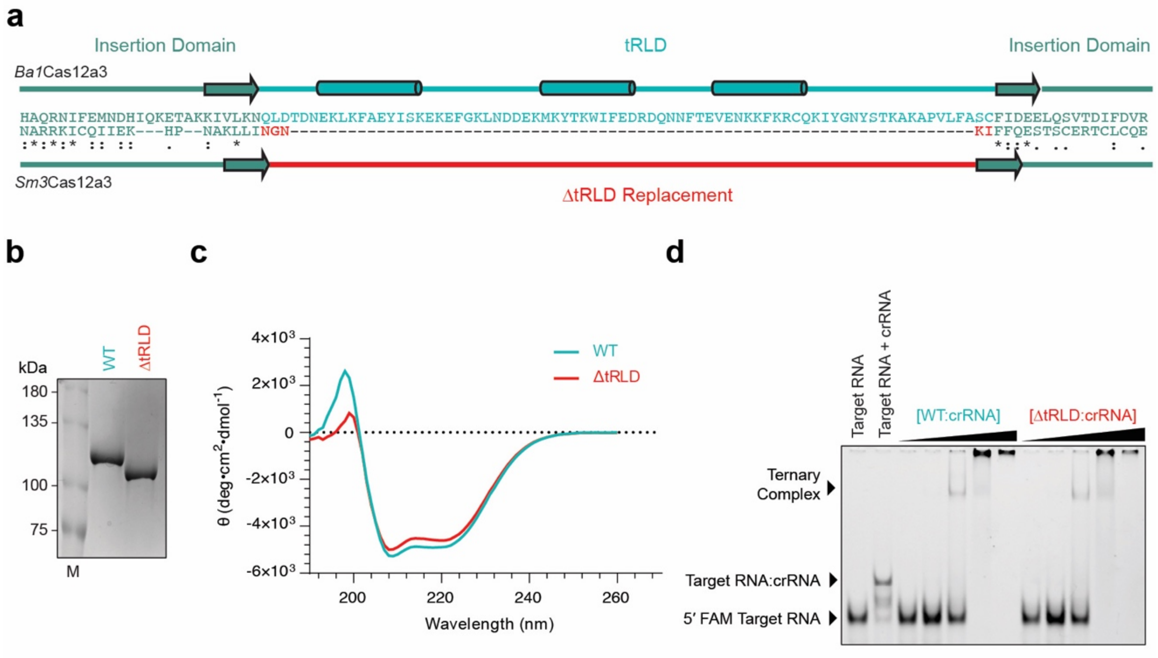

Supplementary Fig. 12. *Ba1*Cas12a3 ΔtRLD maintains its secondary structure and target RNA binding. (a) *Ba1*Cas12a3 and *Sm3*Cas12a3 amino acid alignment. Red indicates the amino acid sequence used to replace the tRNA loading domain (tRLD). **(b)** SDS-PAGE gel of the different purified *Ba1*Cas12a3 constructs. M, marker. **(c)** Circular dichroism (CD) results comparing ΔtRLD to WT *Ba1*Cas12a3 molar ellipticity over wavelength. **(d)** Electrophoretic mobility shift assay of a 5′ FAM RNA target with binary WT or ΔtRLD. For gel source data, see Supplementary Figure 1.

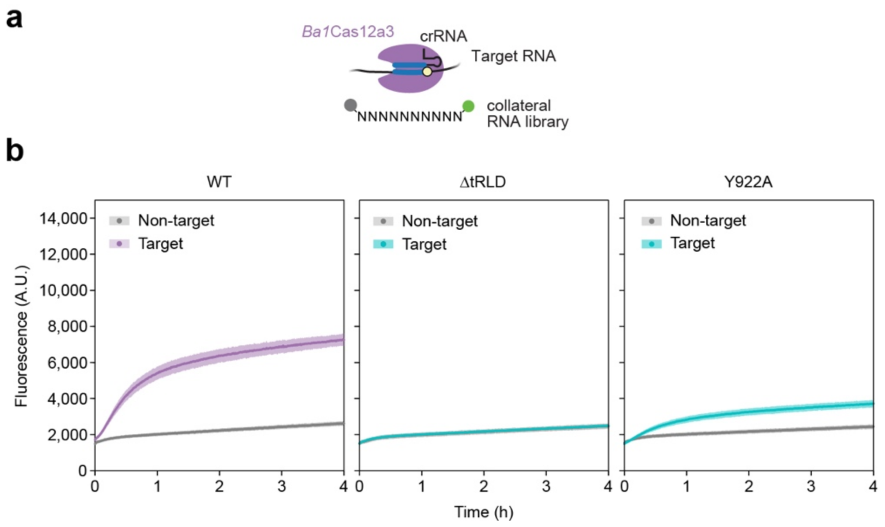

Supplementary Fig. 13. Assessing *in vitro* cleavage of the collateral RNA library by the *Ba1*Cas12a3 mutants ΔtRLD and Y922A. (a) General setup to monitor cleavage of the collateral RNA library by *Ba1*Cas12a3. **(b)** Fluorescence time-course measurements using a target or non-target RNA. Dotted curves and shaded regions represent the mean ± standard deviation of independent cleavage reactions (n = 3 or 4).

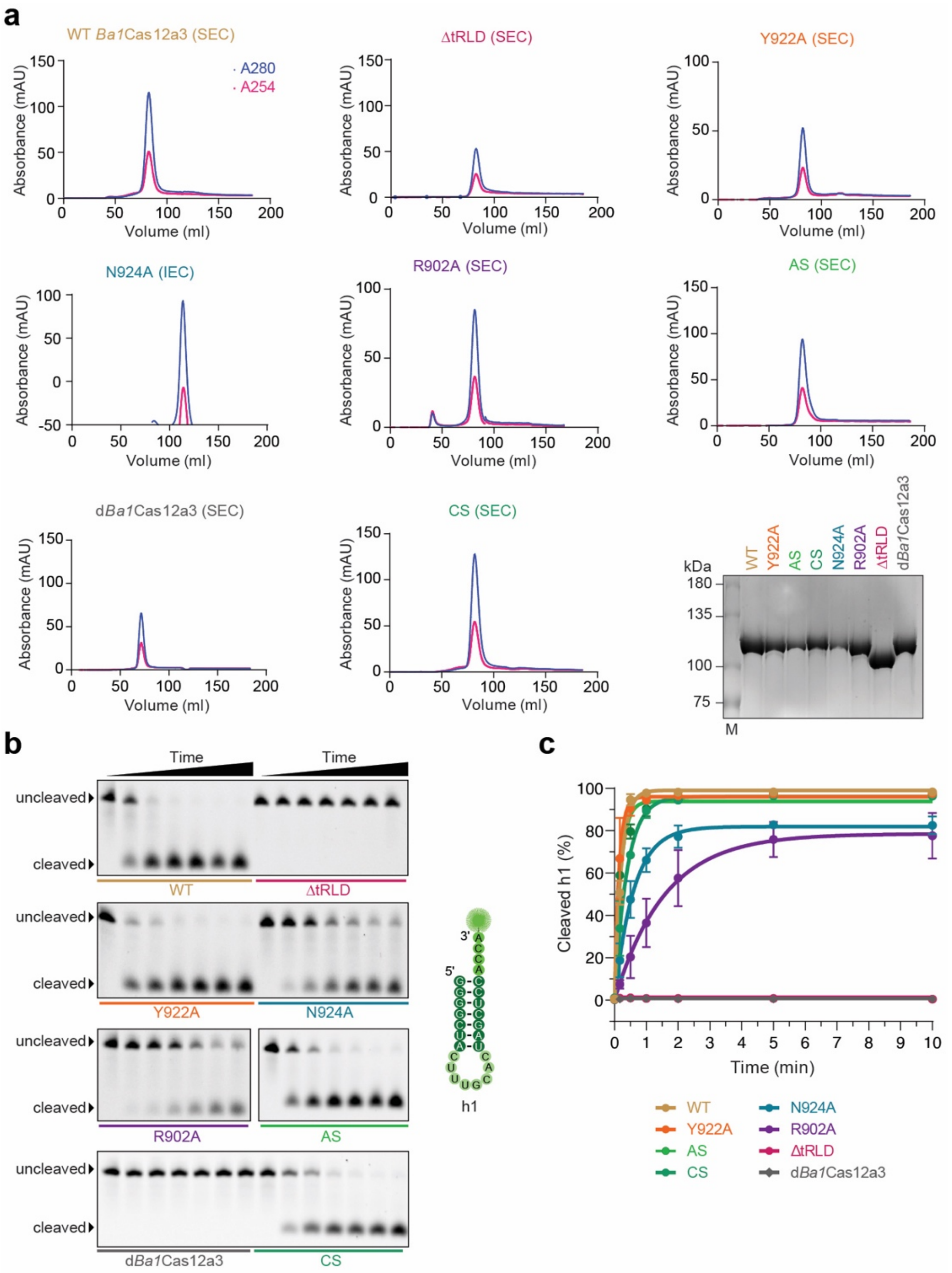

Supplementary Fig. 14. *Ba1*Cas12a3 tRLD and T-arm interacting mutants cleave tRNA mimics at different rates. (a) Size exclusion and ion exchange chromatograms and SDS-PAGE of the *Ba1*Cas12a3 mutants. AS: alanine swap, R251A/N253A/K256A/K257A; CS: charge swap, R251E/N253D/K256E/K257E; d*Ba1*Cas12a3, D712A/E1032A/D1137A. M, marker. **(b)** Collateral cleavage time course assay with the $tRNA^{Ala}$ mimic (h1). **(c)** The percent of h1 cleavage determined using gel densitometry of three independent replicates represented in panel **b**. The rate constants (k_{obs}) reported in **Figure 3f** were determined by fitting the curves of individual

experiments using a one-phase association pseudo-first order equation. Circles and error bars represent the mean ± standard deviation of independent experiments (n = 3). For gel source data, see Supplementary Figure 1.

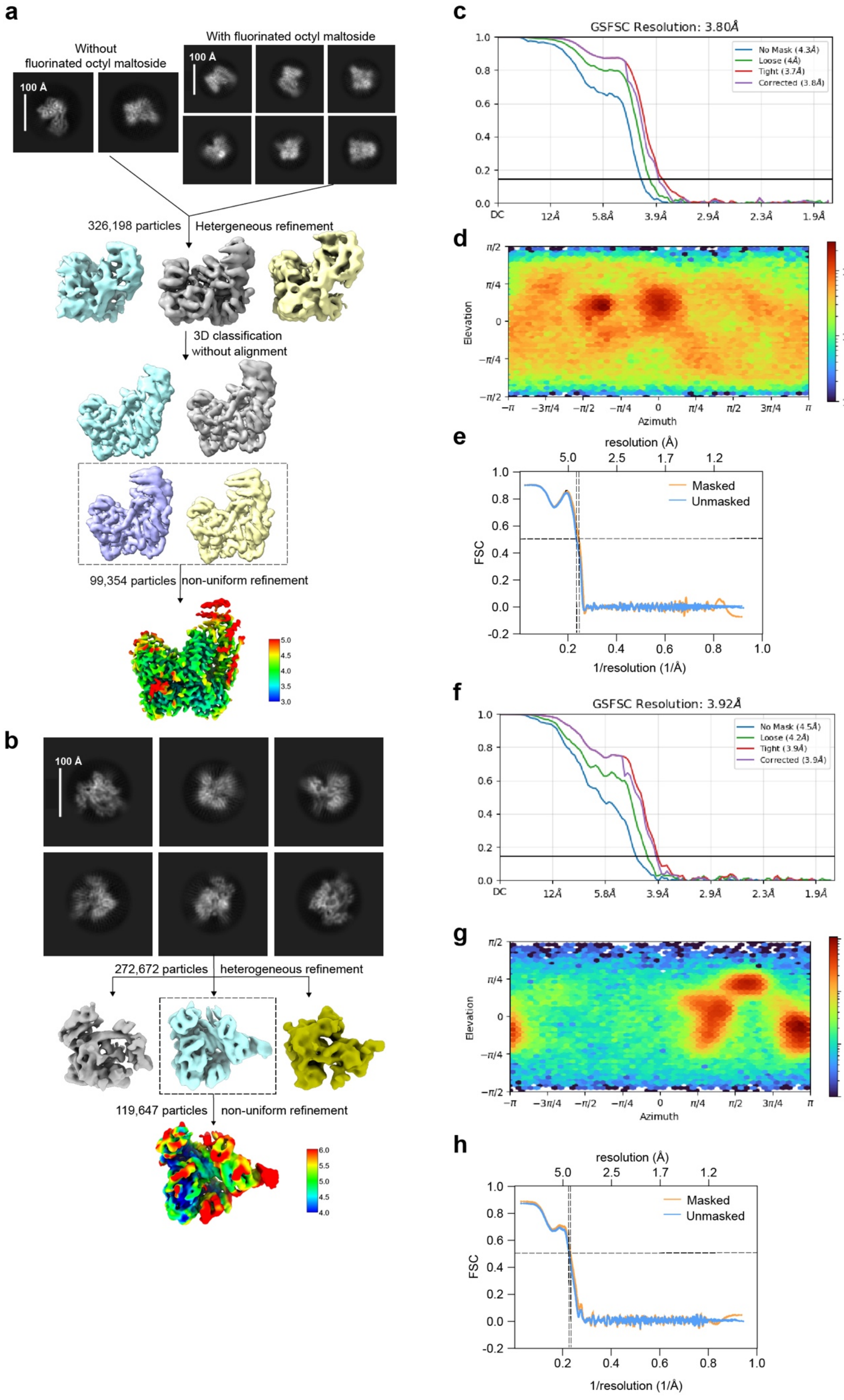
a
Without fluorinated octyl maltoside
With fluorinated octyl maltoside
100 Å
326,198 particles
Hetergeneous refinement
3D classification without alignment
99,354 particles
non-uniform refinement
b
100 Å
272,672 particles
heterogeneous refinement
119,647 particles
non-uniform refinement
c
GSFSC Resolution: 3.80Å
No Mask (4.3Å)
Loose (4Å)
Tight (3.7Å)
Corrected (3.8Å)
d
Elevation
Azimuth
# of images
e
resolution (Å)
Masked
Unmasked
FSC
1/resolution (1/Å)
f
GSFSC Resolution: 3.92Å
No Mask (4.5Å)
Loose (4.2Å)
Tight (3.9Å)
Corrected (3.9Å)
g
Elevation
Azimuth
# of images
h
resolution (Å)
Masked
Unmasked
FSC
1/resolution (1/Å)

Supplementary Fig. 15. Data-processing scheme of *Ba1*Cas12a3 binary and ternary complexes. (a) Data processing flow-chart of *Ba1*Cas12a3 binary complex. W/O:without; FOM: fluorinated octyl maltoside. **(b)** Data processing flow-chart of *Ba1*Cas12a3 ternary complex. 3D heterogeneous refinement separated the dataset into three particle classes (43,544; 119,647; and 109,481 particles, left to right). Only the middle class displayed well-defined guide-target RNA duplex densities, indicative of a fully assembled ternary complex. Subsequent non-uniform refinements in cryoSPARC produced a better-resolved EM map exclusively for the middle class, enabling reliable model building. **(c)** FSC curves for *Ba1*Cas12a3 binary complex. **(d)** Euler plot for *Ba1*Cas12a3 binary complex. **(e)** Map-to-model FSC curves for the *Ba1*Cas12a3 binary complex. **(f)** FSC curves for the *Ba1*Cas12a3 ternary complex. **(g)** Euler plot for *Ba1*Cas12a3 ternary complex. **(h)** Map-to-model FSC curves for *Ba1*Cas12a3 ternary complex.

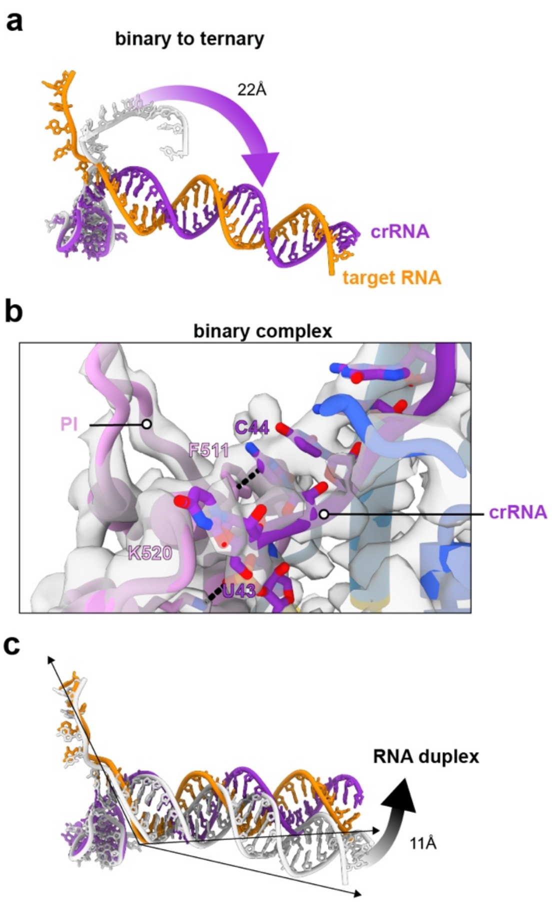

Supplementary Fig. 16. crRNA and target RNA rearrangement during *Ba1*Cas12a3 nuclease activation. (a) Detailed interactions between the PI domain of *Ba1*Cas12a3 and crRNA. **(b)** 3′ of crRNA released from the PI domain forming a guide-target RNA duplex with 5′ target RNA after target RNA recognition. **(c)** Guide-target RNA duplex shifted up to 11 Å after tRNA loading.

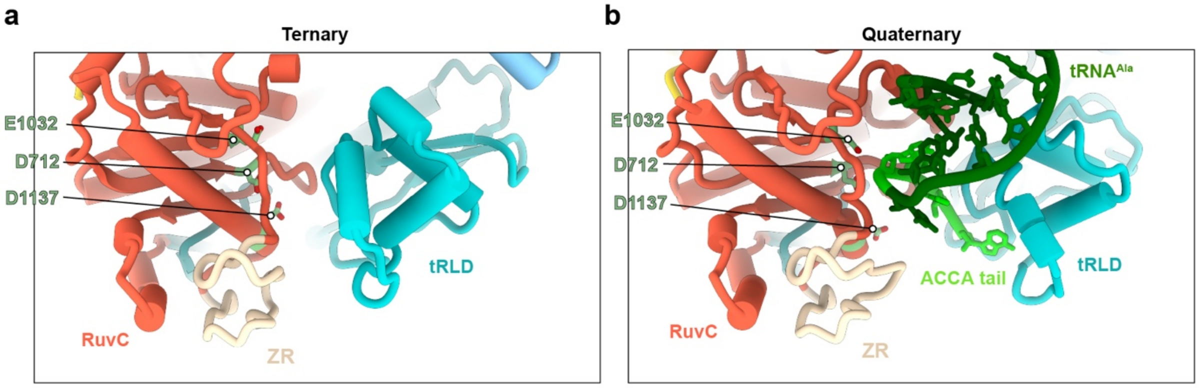

Supplementary Fig. 17. Zoomed-in view of the RuvC and tRLD domains before and after tRNA binding. (a) Close proximity of tRLD and RuvC domains before tRNA loading. The three catalytic residues responsible for substrate cleavage are shown. **(b)** Separation of tRLD and the RuvC domain upon tRNA binding.

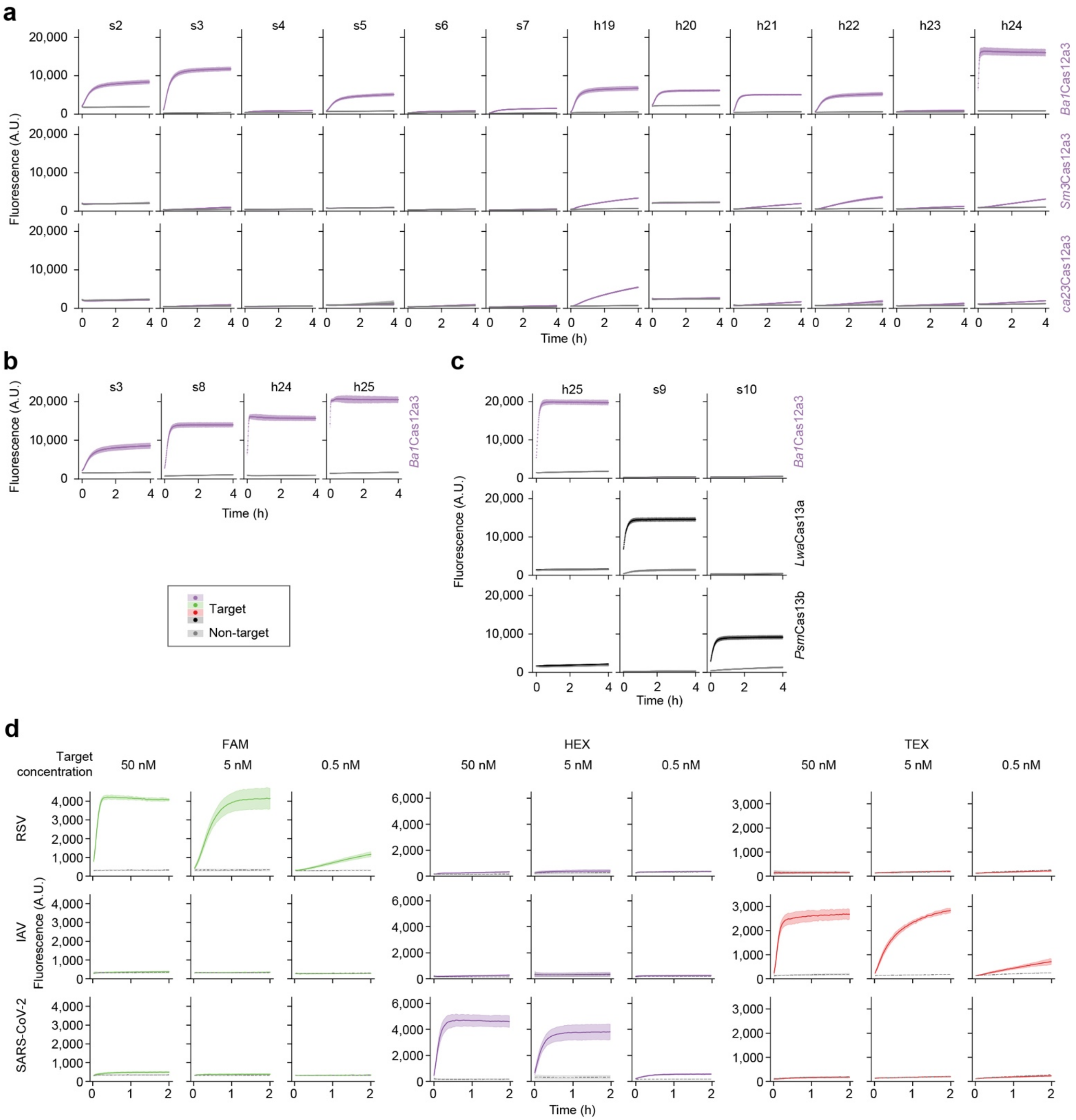
a
s2
s3
s4
s5
s6
s7
h19
h20
h21
h22
h23
h24
Ba1Cas12a3
Sm3Cas12a3
ca23Cas12a3
Fluorescence (A.U.)
20,000
10,000
0
0 2 4
Time (h)
b
s3
s8
h24
h25
Ba1Cas12a3
Fluorescence (A.U.)
Time (h)
Target
Non-target
c
h25
s9
s10
Ba1Cas12a3
LwaCas13a
PsmCas13b
Fluorescence (A.U.)
Time (h)
d
Target concentration
FAM
HEX
TEX
50 nM
5 nM
0.5 nM
RSV
IAV
SARS-CoV-2
Fluorescence (A.U.)
4,000
3,000
2,000
1,000
6,000
0 1 2
Time (h)

e

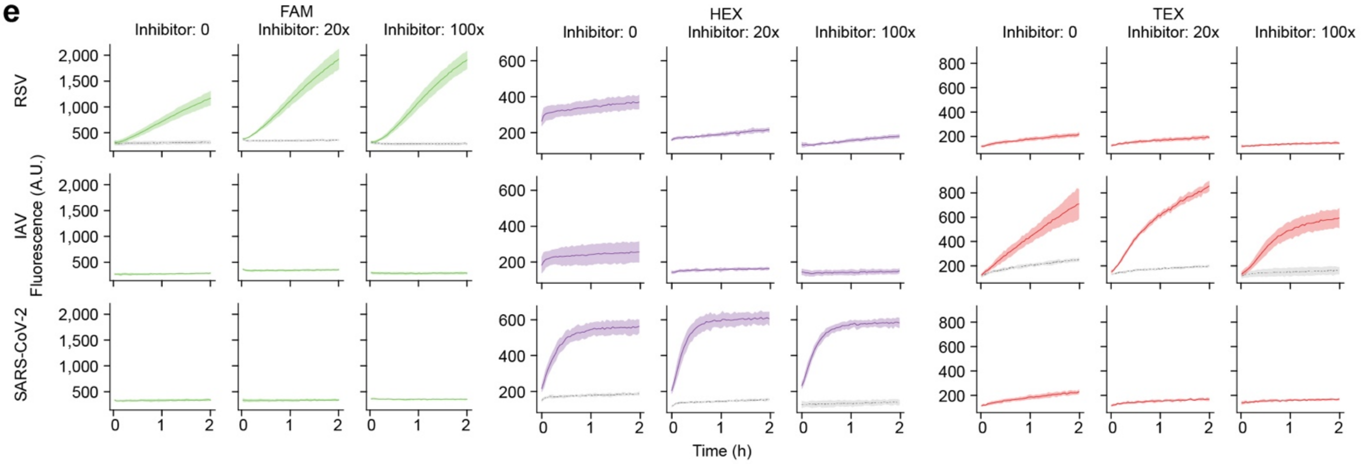

f

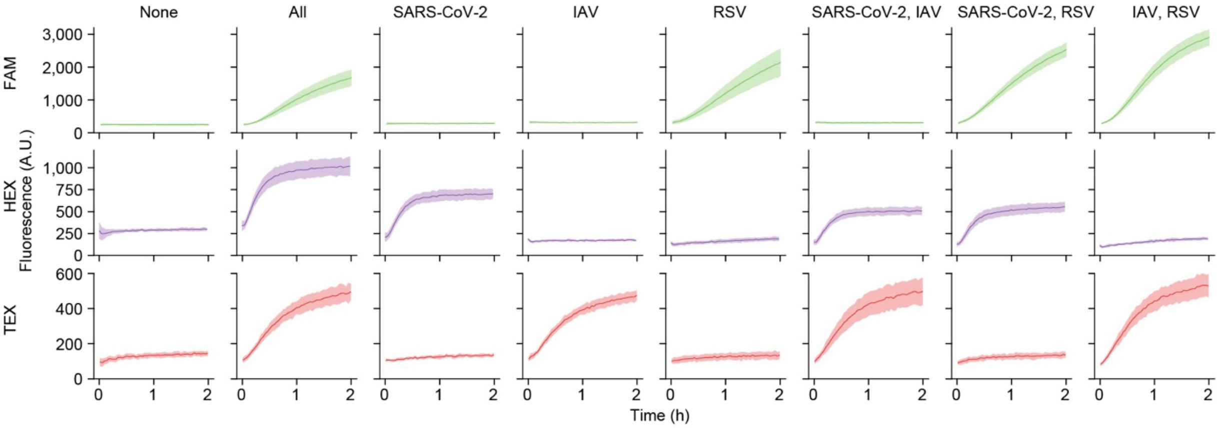

Supplementary Fig. 18. Fluorescence formation over time from the cleavage of tRNA-mimic reporters shown in Figure 5. (a) Time-course of the fluorescence signal generated by cleavage of FAM-FQ-labeled single-stranded (s2–7) and hairpin (h14–19) reporters by *Ba1*Cas12a3, *Sm3*Cas12a3, and *ca23*Cas12a3 nucleases under target and non-target conditions *in vitro*. Scatter plots with error bars represent the mean ± standard deviation of independent experiments (n = 4). The corresponding initial fluorescence formation rates, normalized to each nuclease and the rates calculated as a function of nuclease concentration, are shown in **Figure 5a**. **(b)** Time-course of the fluorescence signal associated with **Figure 5b** (n = 3 or 4). **(c)** Time-course of the fluorescence signal associated with **Figure 5c** (n = 4). **(d)** Time-course of the fluorescence signal associated with **Figure 5e** (n = 4). **(e)** Time-course of the fluorescence signal associated with **Figure 5f** (n = 4). **(f)** Time-course of the fluorescence signal associated with **Figure 5g** (n = 6).
