# Supplementary material for: RNA-triggered Cas12a3 cleaves tRNA tails to execute bacterial immunity
Source: Nature. 2026 Jan 7;649(8099):1312–21. doi: 10.1038/s41586-025-09852-9 (PMC12851939; doi:10.1038/s41586-025-09852-9)
Supplement: Supplementary file 2 — Reporting Summary [file 41586_2025_9852_MOESM2_ESM.pdf]

nature portfolio

Corresponding author(s): Dirk W. Heinz, Ryan N. Jackson, Chase L. Beisel

Last updated by author(s): Oct 23, 2025

Reporting Summary

Nature Portfolio wishes to improve the reproducibility of the work that we publish. This form provides structure for consistency and transparency in reporting. For further information on Nature Portfolio policies, see our Editorial Policies and the Editorial Policy Checklist.

Statistics

For all statistical analyses, confirm that the following items are present in the figure legend, table legend, main text, or Methods section.

n/a	Confirmed	
☐	☒	The exact sample size (*n*) for each experimental group/condition, given as a discrete number and unit of measurement
☐	☒	A statement on whether measurements were taken from distinct samples or whether the same sample was measured repeatedly
☐	☒	The statistical test(s) used AND whether they are one- or two-sided<br>*Only common tests should be described solely by name; describe more complex techniques in the Methods section.*
☒	☐	A description of all covariates tested
☒	☐	A description of any assumptions or corrections, such as tests of normality and adjustment for multiple comparisons
☐	☒	A full description of the statistical parameters including central tendency (e.g. means) or other basic estimates (e.g. regression coefficient) AND variation (e.g. standard deviation) or associated estimates of uncertainty (e.g. confidence intervals)
☐	☒	For null hypothesis testing, the test statistic (e.g. *F*, *t*, *r*) with confidence intervals, effect sizes, degrees of freedom and *P* value noted<br>*Give P values as exact values whenever suitable.*
☒	☐	For Bayesian analysis, information on the choice of priors and Markov chain Monte Carlo settings
☒	☐	For hierarchical and complex designs, identification of the appropriate level for tests and full reporting of outcomes
☒	☐	Estimates of effect sizes (e.g. Cohen's *d*, Pearson's *r*), indicating how they were calculated

Our web collection on statistics for biologists contains articles on many of the points above.

Software and code

Policy information about availability of computer code

Data collection	CFX Maestro v5.3.022.1030, Typhoon FLA 7000, MinKNOW 24.11, Thermo Scientific Smart EPU
Data analysis	Python 3.9, pandas 1.2.0, matplotlib 3.5.2, SAMtools 1.9, ClipKIT, IQ-TREE 2.3.6, warpdemux 0.4.4, dorado 0.8.3, cmsearch, tRNAscan-SE 2.0, BWA-MEM 0.7.17, AlphaFold3, ChimeraX 1.7, ISOLDE, Phenix 1.20.1, DefenseFinder 2.0.1, CRISPRCasFinder 4.2.21, cryoSPARC 4.6, Trimmomatic v0.39

For manuscripts utilizing custom algorithms or software that are central to the research but not yet described in published literature, software must be made available to editors and reviewers. We strongly encourage code deposition in a community repository (e.g. GitHub). See the Nature Portfolio guidelines for submitting code & software for further information.

Data

Policy information about availability of data

All manuscripts must include a data availability statement. This statement should provide the following information, where applicable:

- Accession codes, unique identifiers, or web links for publicly available datasets
- A description of any restrictions on data availability
- For clinical datasets or third party data, please ensure that the statement adheres to our policy

The Illumina-based PFS screen data and the direct RNA Nanopore sequencing reads were deposited at the European Nucleotide Archive (ENA) under accession code PRJEB88250 (https://www.ebi.ac.uk/ena/browser/view/PRJEB88250).

Models and associated cryo-EM maps have been deposited into the PDB database with the following accession codes: Ba1Cas12a3 binary complex (EMD-52275, PDB: 9HLX), Ba1Cas12a3 ternary complex (EMD-52287, PDB: 9HM6), Ba1Cas12a3 quaternary complex at pre-cleavage state (EMD-52285, PDB: 9HM4), and Ba1Cas12a3 quaternary complex at post-cleavage state (EMD-52286, PDB: 9HM5).
Source data are included for graphical results. Raw gel images are included as Supplementary Figure 1.

Research involving human participants, their data, or biological material

Policy information about studies with human participants or human data. See also policy information about sex, gender (identity/presentation), and sexual orientation and race, ethnicity and racism.

Reporting on sex and gender	Not applicable. No human participants, their data, or biological material used in this work.
Reporting on race, ethnicity, or other socially relevant groupings	Not applicable. No human participants, their data, or biological material used in this work.
Population characteristics	Not applicable. No human participants, their data, or biological material used in this work.
Recruitment	Not applicable. No human participants, their data, or biological material used in this work.
Ethics oversight	Not applicable. No human participants, their data, or biological material used in this work.

Note that full information on the approval of the study protocol must also be provided in the manuscript.

Field-specific reporting

Please select the one below that is the best fit for your research. If you are not sure, read the appropriate sections before making your selection.

☒ Life sciences ☐ Behavioural & social sciences ☐ Ecological, evolutionary & environmental sciences

For a reference copy of the document with all sections, see nature.com/documents/nr-reporting-summary-flat.pdf

Life sciences study design

All studies must disclose on these points even when the disclosure is negative.

Sample size	Quantitative experiments involved a sample size of at least three, the minimal number needed to perform statistical analyses. The exact sample size is indicated in each figure legend.
Data exclusions	No data were excluded from the analyses.
Replication	All measurements were performed in at least three biological replicates, unless stated otherwise. All replicates were successful.
Randomization	No randomization was performed, as is common in molecular biology and microbiology studies. In some cases, values were normalized to a control condition in the same experiment, such as the PFS library under non-targeting conditions (e.g., Fig. 1c), CFUs under non-targeting conditions (e.g., Fig. 1d) or the maximum enzymatic activity in an in vitro cleavage assay (e.g., Fig. 1f), to reduce between -experiment variability independent of the specific sample.
Blinding	No blinding was performed, as is common in molecular biology and microbiology studies. A major rationale is that the process of experimental design, conducting an experiment, and analyzing the results is conducted by a single researcher, and involving others to blind the results creates an additional burden on personnel and creates the potential of labeling errors. Instead, results are confirmed through biological replicates and parallel assays to ensure consistency, such as testing Cas12a3 nucleases in E. coli and in vitro (e.g., Figs. 1d-f).

Reporting for specific materials, systems and methods

We require information from authors about some types of materials, experimental systems and methods used in many studies. Here, indicate whether each material, system or method listed is relevant to your study. If you are not sure if a list item applies to your research, read the appropriate section before selecting a response.

Materials & experimental systems

n/a	Involved in the study
☒	☐ Antibodies
☒	☐ Eukaryotic cell lines
☒	☐ Palaeontology and archaeology
☒	☐ Animals and other organisms
☒	☐ Clinical data
☒	☐ Dual use research of concern
☒	☐ Plants

Methods

n/a	Involved in the study
☒	☐ ChIP-seq
☒	☐ Flow cytometry
☒	☐ MRI-based neuroimaging

Plants

Seed stocks	No plants used in this study.
Novel plant genotypes	No plants used in this study.
Authentication	No plants used in this study.
